# Influence of Heterologous and Homologous Vaccines, and Their Components, on the Host Immune Response and Protection Against Experimental Caprine Paratuberculosis

**DOI:** 10.3389/fvets.2021.744568

**Published:** 2022-01-05

**Authors:** Noive Arteche-Villasol, Daniel Gutiérrez-Expósito, Natalia Elguezabal, Iker A. Sevilla, Raquel Vallejo, José Espinosa, María del Carmen Ferreras, Julio Benavides, Valentín Pérez

**Affiliations:** ^1^Departamento de Sanidad Animal, Facultad de Veterinaria, Universidad de León, León, Spain; ^2^Departamento de Sanidad Animal, Instituto de Ganadería de Montaña (CSIC-ULE), León, Spain; ^3^Departamento de Sanidad Animal, NEIKER-Instituto Vasco de Investigación y Desarrollo Agrario, Derio, Spain

**Keywords:** vaccination, paratuberculosis, tuberculosis, HIMB, adjuvant, cross-protection

## Abstract

Vaccination against paratuberculosis, a chronic disease of ruminants caused by *Mycobacterium avium* subsp. *paratuberculosis* (*Map*), has been considered as the most effective control method. However, protection is incomplete, and the mechanisms operating in the response of the animals to vaccination are not fully understood. Therefore, this study analyzed the immune response and the effects on protection against *Map* infection, elicited by paratuberculosis (Silirum®) and tuberculosis (heat-inactivated *M. bovis* [HIMB]) vaccines and their components in a caprine experimental model. Fifty goat kids were divided into 10 groups (*n* = 5) according to their vaccination (Silirum®, HIMB and nonvaccinated), immunization (inactivated bacteria or adjuvant), and/or infection. Oral challenge with *Map* was performed 45 days postvaccination/immunization (dpv), and animals were euthanized at 190 dpv. Peripheral immune response and proportion of lymphocyte subpopulations were assessed monthly by enzyme-linked immunosorbent assay and flow cytometry analysis, respectively. Local immune response, proportion of tissue lymphocyte subpopulations, *Map* detection (polymerase chain reaction), and histological examination were conducted in gut-associated lymphoid tissues. All infected groups developed paratuberculosis granulomatous lesions despite vaccination or immunization. The Silirum® and HIMB-vaccinated groups showed a considerable lesion reduction consistent with a significant peripheral cellular and humoral immune response. Besides, a lower number of granulomas were observed in groups immunized with inactivated bacteria and adjuvants in comparison to nonvaccinated and infected group. However, despite not being significant, this reduction was even higher in adjuvant immunized groups, which developed milder granulomatous lesion with no detectable peripheral immune responses associated with immunization. No changes in the peripheral and local proportion of lymphocyte subsets or local immune response were detected in relation to either vaccination/immunization or infection. Despite that paratuberculosis and tuberculosis vaccination showed a partial and cross-protection against *Map* infection, respectively, only histological examination could assess the progression of infection in these animals. In addition, the pattern observed in the reduction of the lesions in adjuvant immunized groups suggests the possible involvement of a nonspecific immune response that reduces the development of granulomatous lesions.

## Introduction

Paratuberculosis is a chronic and debilitating disease of ruminants caused by *Mycobacterium avium* subsp. *paratuberculosis* (*Map*) (1) and responsible for significant global economic losses estimated at approximately US $12.61 million and $364.31 million in Spanish and European Union dairy cattle, respectively (2, 3). *Map* is a ubiquitous bacterium transmitted among the livestock at an early age through the fecal–oral route, although clinical manifestations may appear several years after infection (4). Initial protective immune response against *Map* has been related to a strong cell-mediated immune response characterized by the release of the proinflammatory cytokine interferon γ (IFN-γ), the formation of granulomas, and the clearance of the mycobacteria (5, 6). In this sense, *Map*-infected animals can show a variety of lesions that have been shown to be closely related to the different phases of the disease. Early paratuberculosis lesions are characterized by small and well-demarcated granulomas, named *focal* or *multifocal* forms, located within the intestinal lymphoid tissue that can be also seen in adult animals, where they are considered as forms of latency or resistance; disruption of the protective cell-mediated immune response leads to the development of *diffuse* lesions and the evolution toward a widespread granulomatous enteritis and the occurrence of clinical signs (7, 8). Pathological methods for paratuberculosis lesion characterization have been successfully applied in previous works as a reliable indicator of the presence of *Map* infection and the form of the disease shown by the infected animals (9–11).

The long incubation period and the ability of *Map* to persist in the environment hamper control programs based on hygiene-management measures or early diagnosis and culling of positive animals (12, 13). For this reason, vaccination with commercially available heat-killed vaccines has been considered as the most cost-effective measure for the control of paratuberculosis, as its use reduces the incidence of the disease within the herd (14, 15). Vaccination leads to a reduction of both the colonization of intestinal tissues and the number of clinically affected animals, achieving a decrease by approximately 90% in the occurrence of *Map*-shedding animals (16–18).

However, the mechanisms that might be involved in the protection associated with vaccination are yet not fully understood (12, 13). Protection conferred by vaccines against *Map* infection has been correlated with an early and strong cell-mediated immune response (19–21), despite the fact that there is a low percentage of vaccination failure, where some vaccinated animals could remain highly infectious and develop severe lesions (16, 22, 23).

Most paratuberculosis heat-killed vaccines are based on *Map* 316F strain, which has demonstrated reduced virulence (tissue bacterial burden) during experimental infection of calves with this live isolate likely due to continuous *in vitro* passages (24). In this sense, subcutaneous immunization of sheep with heat-killed *Map* 316F strain alone did not show significant cell-mediated and humoral immune response (25). Supplementation with mineral oil adjuvants is a core principle in order to enhance the immunogenicity of antigens and their continuous liberation in order to promote a robust immune response through the mount of a strong local inflammation at the site of injection (26). In a previous experiment, significant variations in the immune response were found when paratuberculosis vaccines made with different adjuvants were subcutaneously administered to sheep (25). On the other hand, the sole administration of adjuvants has been shown to elicit an unspecific immune response whose protective effect has not been evaluated in detail (27–29). In the case of paratuberculosis vaccines, the effect that each component could have in a protective response against *Map* infection has not been fully elucidated.

Besides, *Map* shares a high number of common antigens with related mycobacteria such as *Mycobacterium bovis* (*Mbv*) or *Mycobacterium caprae*, responsible for tuberculosis in ruminants, a fact that is beyond the cross-reactions that appear when immunological-based tests are used for tuberculosis diagnosis in paratuberculosis infected or vaccinated animals (30, 31). This also could be associated with the significant reduction of tuberculosis-related lesions achieved after paratuberculosis vaccination in goats and calves (32, 33). Despite this fact, the effect of immunization with tuberculosis-related mycobacteria on the development of paratuberculosis is yet unknown.

Therefore, the objective of this study was to analyze the effect of homologous or heterologous vaccination of goats, and a subsequent challenge with *Map*, on the immune response and protection and evaluate whether the different components from these vaccines (inactivated bacteria or adjuvant) could participate in the response of the vaccinated host against *Map*.

## Materials and Methods

### Ethics Statement

All the experimental procedures were carried out according to European (86/609) and Spanish laws (R.D. 223/1988, R.D. 1021/2005, R.D. 53/2013) and approved by the local government following the report and suggestions from the Subcommittee on Animal Experiments and Welfare of the University of León (ULE) (OEBA-ULE-016-2017).

### Vaccines, Immunization Products, and Challenge Inoculum

Silirum® commercial vaccine against paratuberculosis and its components (the Montanide™ adjuvant and the *Map* 316F strain included in the vaccine) were prepared separately and shipped by the manufacturer, CZ Vaccines (Porriño, Spain), whereas the heat-inactivated *Mbv* vaccine (HIMB) and its components (a Montanide™ adjuvant and the 1403 *Mbv* strain) were prepared by NEIKER as previously described (34). Briefly, each dose of HIMB vaccine (1 mL) consisted of an aqueous suspension of heat-inactivated 10^7^ colony-forming units (CFUs) of *Mbv* 1403 NEIKER's strain (84–85°C for 45 min) emulsified in Montanide™ ISA 50 V 2 adjuvant (Seppic, France). Furthermore, each inactivated bacteria and adjuvant immunization products were adjusted to the same dose administered in Silirum® and HIMB vaccines. Thus, each dose (1 mL) of inactivated bacteria (10^9^ CFUs of *Map* 316F and 10^7^ CFUs of *Mbv* 1403 strains) was diluted in phosphate-buffered saline (PBS), whereas each dose of Montanide™ adjuvant (1 mL) (used in Silirum® and HIMB vaccines) was emulsified in PBS.

Besides, bovine *Map* 764 strain was prepared for the challenge as previously described by Fernández et al. (11). Briefly, *Map* 764 strain was grown on Middlebrook 7H9 broth enriched with 10% oleic acid–albumin–dextrose–catalase (OADC) and 2 mg · L^−1^ Mycobactin J (7H9 OADC MJ) for 3 weeks at 37 ± 1°C. Then, cultures were harvested by centrifugation at 3,000*g* for 10 min, and bacterial pellets were washed twice and resuspended in PBS. In order to disrupt bacterial clumps, resultant suspension was passed up and down through a 27-gauge needle several times and vortexed. Bacterial concentration was estimated by optical density (O.D.) and CFU estimation of 10-fold serial dilutions plated onto agar-solidified 7H9 OADC MJ. Finally, suspensions were adjusted to 1.2 × 10^10^ CFUs · mL^−1^ and maintained at 4°C throughout all the challenge period (2 weeks), and bacterial clumps were again disrupted before oral inoculation as mentioned previously.

### Experimental Design

A total of 50 female Murciano–Granadina breed goat kids of age 1 month were used in this study. Animals were selected from a flock without clinical signs and tested negative to paratuberculosis and tuberculosis in the last 10 years. Moreover, no positive reactors were identified in the annual official tuberculosis eradication campaigns, based on intradermal skin test, conducted by the regional animal health authorities during the last 5 years. The paratuberculosis- and tuberculosis-free status of the experimental animals was confirmed using antibody enzyme-linked immunosorbent assay (ELISA) against *Map* (ID Screen® Paratuberculosis indirect, IDVet, Gabrels, France) and *Mbv* (INgezim Tuberculosis DR, Eurofins Technology, Madrid, Spain) and the IFN-γ release test (Bovigam® *Mbv* IFN-γ test for cattle, Thermo Fisher Scientific, Waltham, USA). After an adaptation period of 15 days in the facilities of the Instituto de Ganadería de Montaña (IGM-ULE) in León (Spain), goat kids were randomly allocated in separated pens, subjected to standard management practices, and their health and welfare status was checked daily.

**Figure 1:**
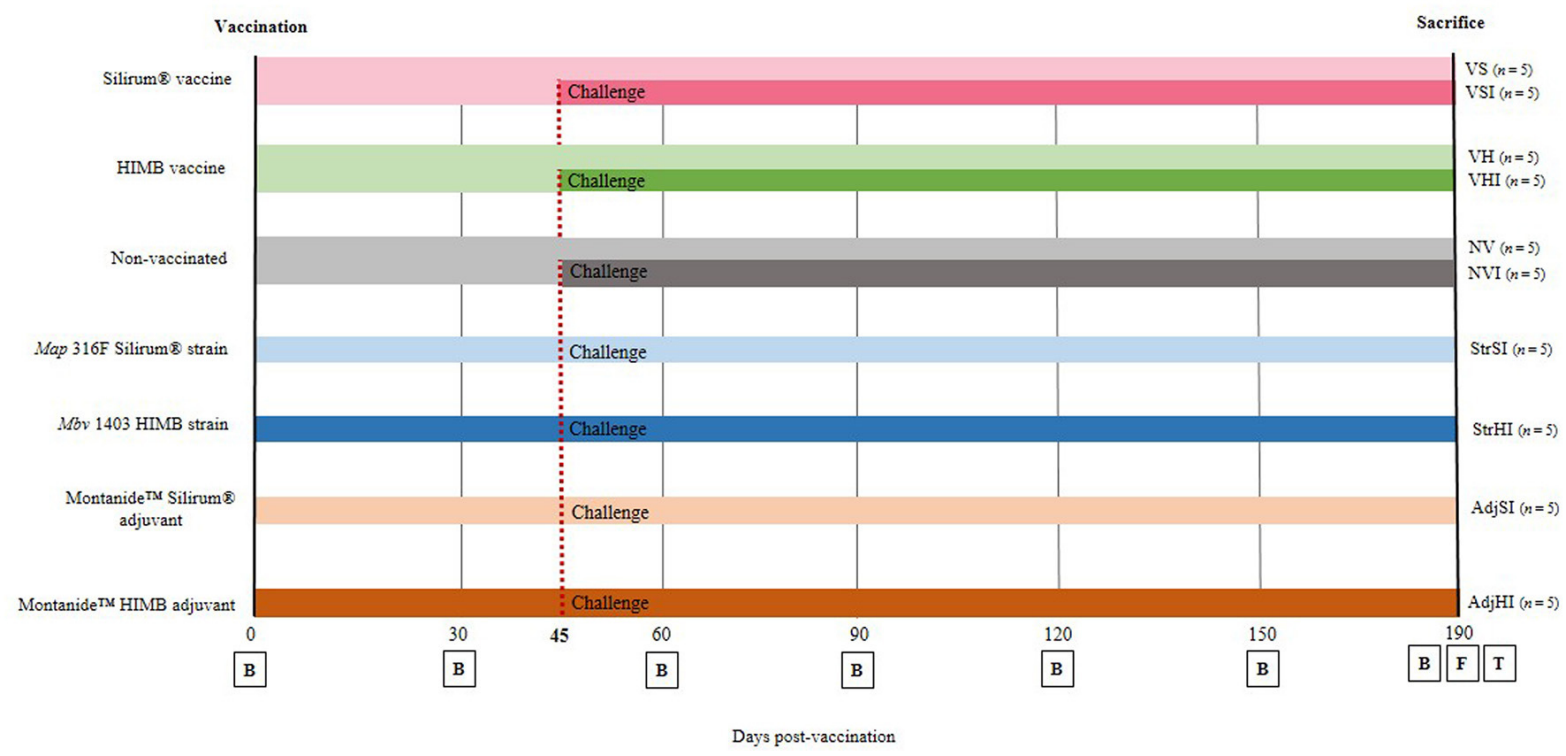
Experimental design scheme. Goats were divided into seven groups and vaccinated with different components at 0 days postvaccination (dpv). From then until sacrifice (190 dpv), blood samples (B) were taken at 30-day interval. Five goats from each group were orally challenged with *Map* 764 strain at 45 dpv and divided into 10 groups: NV, nonvaccinated and noninfected; NVI, nonvaccinated and infected; VS, Silirum® vaccinated and noninfected; VSI, Silirum® vaccinated and infected; VH, HIMB vaccinated and noninfected; VHI, HIMB vaccinated and infected; StrSI, *Map* 316F Silirum® strain immunized and infected; StrHI, *Mbv* 1403 HIMB strain immunized and infected; AdjSI, Montanide™ Silirum® adjuvant immunized and infected; AdjHI, Montanide™ HIMB adjuvant immunized and infected. Finally, at 190 dpv, feces (F) were collected, and culling and complete necropsies for tissue (T) sample collection were carried out.

At the beginning of the study, and according to the vaccination and/or infection protocol to be followed, goats were classified into 10 groups (*n* = 5) and distributed in different pens in order to prevent direct contact between groups according to the following scheme (Figure 1):

VS: Silirum® vaccinated and noninfected

VSI: Silirum® vaccinated and infected

VH: HIMB vaccinated and noninfected

VHI: HIMB vaccinated and infected

StrSI: immunized with *Map* 316F Silirum® strain and infected

StrHI: immunized with *Mbv* 1403 HIMB strain and infected

AdjSI: immunized with Montanide™ Silirum® adjuvant and infected

AdjHI: immunized with Montanide™ HIMB vaccine adjuvant and infected

NV: nonvaccinated and noninfected

NVI: nonvaccinated and infected

No uninfected animals were included in groups immunized with inactivated bacteria or adjuvants, because of the fact that the study was focused on the evaluation of the effect of the components of the vaccines on protection, after *Map* infection, and this would involve the inclusion of a large number of animals and groups that would hinder the study.

Vaccination was performed at the beginning of the experiment, on day 0, by a subcutaneous injection in the brisket with 1 mL of Silirum® vaccine, 10^9^ *Map* 316F Silirum® vaccine strain CFUs, 1 mL of Montanide™ Silirum® adjuvant (CZ Vaccines, Porriño, Spain), 1 mL of HIMB vaccine, 10^7^ *Mbv* 1403 HIMB vaccine strain CFUs, and Montanide™ HIMB vaccine adjuvant, whereas 10 animals remained as the control group (nonvaccinated) and were inoculated subcutaneously with 1 mL of PBS (Figure 1).

Forty-five days postvaccination (dpv), animals were challenged orally (Figure 1) using an automatic syringe with a total amount of 1.2 × 10^10^ *Map* 764-CFUs diluted in 40 mL of PBS as previously described by Fernández et al. (11), whereas 40 mL of PBS was administered orally to noninfected animals at the same time. Throughout the experimental trial, all goats were monitored daily for clinical signs and sampled every 30 days until sacrifice (Figure 1). At 190 dpv, complete necropsies and postmortem sampling were performed on all goats after being humanely euthanized by deep sedation with xylazine (XILAGESIC^®^, Laboratorios Calier, Barcelona, Spain) and a subsequent intravenous injection of T61® (MSD Animal Health, Salamanca, Spain) followed by exsanguination (Figure 1).

### Sample Collection

Blood samples were monthly collected from the jugular vein into Vacutainer tubes with lithium heparin (Becton Dickinson and Company, UK) and without anticoagulant (Becton Dickinson and Company, UK). Then, heparinized samples were processed immediately for IFN-γ release test in response to protein derivative of avian (PPDa) and bovine (PPDb) antigens at 0, 30, 60, 90, 120, 150, and 190 dpv (Figure 1). At the same time points, heparinized blood was also collected for peripheral blood mononuclear cell (PBMC) isolation (35) and their subsequent characterization by flow cytometry. Besides, nonheparinized samples were processed for *Map* (ID Screen® Paratuberculosis indirect, IDVet, Gabrels, France) and *Mbv*-specific (INgezim Tuberculosis DR, Eurofins Technology, Madrid, Spain) antibody determination tests at 0, 30, 60, 90, 120, 150, and 190 dpv (Figure 1).

Fecal samples from each goat were collected separately into disposable plastic gloves directly from the rectum at 190 dpv, prior to the euthanasia of the animal, and frozen at −20°C until processing for *Map* detection through bacteriological culture.

Animals were euthanized at day 190 dpv and a regulated, orderly, and complete necropsy was performed. After gross examination of the viscera, samples from ileum (proximal, medium, and distal zones), jejunum (proximal, medium, and distal zones), ileocecal valve, and jejunal Peyer patches (at least three patches from each zone: proximal, medium, and distal), together with mesenteric, jejunal, and ileocecal lymph nodes, were taken into buffered formol saline fixative for histological examination. In addition, samples of distal ileum, jejunal Peyer patches, and mesenteric lymph node were also collected and stored at −20°C for *Map* isolation by culture and detection by real-time quantitative polymerase chain reaction (qPCR) as well as processed immediately for leukocyte isolation in order to characterize lymphocyte subpopulation by flow cytometry and also to perform IFN-γ release test in response to PPDa and PPDb antigens.

### Isolation of PBMCs and Tissue Leukocytes

PBMCs were isolated as previously described (35). Briefly, 30 mL of heparinized peripheral blood was centrifuged, and PBMCs were isolated by gradient centrifugation using Lymphoprep™ (STEMCELL Technologies®, Cologne, Germany). Resultant PBMCs were washed three times with PBS, and cell suspensions were resuspended in supplemented RPMI1640 medium + GlutaMax™ (Gibco, Paisley, UK) and counted in a Neubauer chamber and adjusted at a final concentration of 10^6^ cells · mL^−1^. Intestinal samples and mesenteric lymph nodes were collected and washed in PBS during the necropsy, put into individual falcon tubes containing 30 mL of sterile supplemented RPMI1640 medium, and processed in the laboratory within 30 min after collection. For tissue lymphocyte isolation, 5 cm of ileum and jejunal Peyer patches were longitudinally opened showing the mucosa and washed with PBS until fecal remains were eliminated, whereas pericapsular fat was removed from the lymph nodes. The excess tissue around each Peyer patch was removed, and the mucosa from ileum and Peyer patches were scraped and minced. Besides, 50 mg of mesenteric lymph node tissue was cut into small pieces and chopped using a scalpel blade. Minced tissue was suspended in 11 mL of PBS with EDTA (2 mM) and processed with a stomacher blender (Masticator, IUL) for 2.5 min. Then, 10 mL from the upper homogenized portion was passed through 40-μm filter (Thermo Fisher Scientific, Madrid, Spain), and resultant suspension was layered in an equal volume of Lymphoprep™ and centrifuged at 800 *g* for 30 min with no stop or acceleration. Cells from the interface layer were washed three times with PBS/EDTA, counted in a Neubauer chamber and resuspended in supplemented RPMI1640 at a final concentration of 10^6^ cells · mL^−1^.

Cell viability determined by trypan blue dye exclusion was usually >90% for both PBMCs and tissue lymphocytes (data not shown).

### Peripheral and Local Cell-Mediated and Peripheral Humoral Immune Response

Whole heparinized peripheral blood samples taken at 0, 30, 60, 90, 120, 150, and 190 dpv were processed within 3 h of collection. For each animal, three wells of a 24-well tissue plate (Thermo Fischer Scientific, Rochester, NY) were filled with 1.5 mL of blood each and were either mixed with 100 μL of sterile PBS (negative control) or stimulated with 100 μL of PPDa or PPDb (CZ Vaccines, Porriño, Spain) at a final concentration of 30 μg · mL^−1^ diluted in sterile PBS (11). After 22 h of stimulation, plates were centrifuged at 750 *g* for 15 min, and plasma was collected and stored at −20°C until tested.

For the analysis of the cell-mediated local immune response, a total of 2 × 10^6^ mononuclear leukocytes isolated from ileum, Peyer patches, and mesenteric lymph node were seeded per well into three wells each of a 24-well tissue plate and stimulated as described for whole blood. After 22 h of stimulation, plates were centrifuged at 750 *g* for 15 min, and supernatants were collected and stored at −20°C until tested.

IFN-γ production in these assays was assessed by duplicate using commercial ELISA for bovine IFN-γ following manufacturer's instructions (Bovigam® *Mbv* IFN-γ test for cattle, Thermo Fisher Scientific, Waltham, USA), and absorbance values were measured spectrophotometrically using an ELX800 ELISA reader (Bio-Tek Instruments) at 450 nm. The O.D. values were adjusted by dividing the plasma or supernatant O.D. value by the negative control O.D. value of each plate in order to prevent interplate variations. Results were expressed as avian and bovine index values by means of the quotient between the mean O.D. of PPDa or O.D. of PPDb-stimulated plasma, respectively, and the mean O.D. of the negative control plasma (11, 36).

Nonheparinized blood samples were allowed to clot and retract, and serum was stored at −20°C until used. Each serum was tested for specific antibodies against *Map* (37) and *Mbv* (38) using commercial ELISA tests (ID Screen® Paratuberculosis indirect, IDVet, Gabrels, France; and INgezim Tuberculosis DR Eurofins Technology, Madrid, Spain, respectively) following manufacturer's instructions. Results were expressed as the *S/P* ratio of *Map* and *Mbv* antibodies calculated by dividing the corrected O.D. of the sample by the corrected O.D. of the positive control and multiplying by 100 (39).

### Real-Time Quantitative Detection of *Map*

DNA was extracted from 50 mg of distal ileum, jejunal Peyer patches, and mesenteric lymph node by using Maxwell® 16 Tissue DNA Purification Kit (Promega, WI, USA) with the Maxwell 16 Instrument (Promega) following manufacturer's instructions. Thereupon, DNA was quantified using QuantiFluor™ ONEdsDNA System kit (Promega, WI, USA) and Quantus™ Fluoremeter (Promega, WI, USA). Extracted DNA was diluted at 50 ng · μL^−1^ and stored at −20°C until qPCR was performed. Genomic DNA from 2 × 10^8^ *Map* CFUs and that from 50 mg of tissues of a noninfected animal were extracted and quantified to generate a standard curve.

Detection of *Map* IS*900* sequence was performed as previously described by Arteche-Villasol et al. (40). In addition, *Map*-DNA quantification and qPCR analytical sensitivity were assessed by the construction of a 10-fold diluted standard curve using *Map*-genomic DNA ranging from 1,000 pg to 0.001 pg/reaction mixed with 100 ng/reaction of tissue DNA from a noninfected animal. Samples were considered as positive when the dissociation peak (*Tm*) was 89.1 ± 1.5°C and threshold cycles (*Ct*) were ≤ 37 (41, 42). The qPCR results were analyzed using 7500 Software v2.0.6 (Applied Biosystems™). *Map*-DNA quantity (pg) of each well was calculated by interpolation of their *Ct* values with the standard curve as previously described (43), and the mean quantity was calculated from both duplicates.

### Tissue and Fecal Culture of *Map*

Distal ileum, jejunal Peyer patches, and mesenteric lymph node from each animal were tested individually, whereas fecal samples were pooled into groups of three goats each. Ileum and Peyer patch segments (12 cm) and 2 grams of mesenteric lymph node were processed as described for tissue leukocyte isolation and following methods previously described (44). Briefly, 2 g of each tissue and fecal samples pools were decontaminated with 38 mL of hexadecylpyridinium chloride and homogenized in a stomacher blender (Masticator, IUL) for approximately 15 s. After 18 h of decontamination, 200 μL of the suspension was used to inoculate two tubes containing Herrold's egg yolk medium supplemented with sodium pyruvate and mycobactin J (MJ) and two tubes containing 7H9 OADC supplemented with MJ, penicillin, amphotericin, and chloramphenicol. Cultures were incubated at 37°C ± 1°C, and growth was checked by examination under a stereoscopic microscope after 8, 12, 16, and 20 weeks postinoculation. Cultures were considered positive if one or more characteristic *Map* colonies were observed in any tube. Colonies isolated on both mediums were confirmed by a real-time multiplex PCR detecting IS*900* and IS*Map02 Map* sequences (45).

### Flow Cytometric Analysis of PBMCs and Tissue Leukocytes

Single-color flow cytometry analysis was carried out for phenotypic characterization of PBMCs isolated at 0, 30, 60, 90, 120, 150, and 190 dpv and mononuclear leukocytes isolated from distal ileum, jejunal Peyer patches, and mesenteric lymph node. A total number of 2 × 10^5^ cells per well were seeded in a 96-well plate (Thermo Fisher Scientific, Roskilde, Denmark) and incubated with primary antibodies against lymphocyte surface markers detailed in Table 1 for 1 h at 4°C. Afterward, cells were washed twice with PBS and incubated with appropriate conjugated secondary antibodies for 1 h at 4°C (Table 1). Finally, cells were fixed with 1% of CellFIX™ (Becton Dickinson and Company, Erembodegem, Belgium) until analyzed. Sample acquisition of 10,000 events was performed using a flow cytometer (MACSQuant, Miltenyi Biotec®), where events were gated during the acquisition analysis as previously described elsewhere (35) to discard the presence of air and doublets. Then, analysis of data was carried out using the MACSQuantify10 Software™ (Miltenyi Biotec®), and results were expressed as percentage of positive cells.

**Table 1:** Primary and secondary antibodies used in flow cytometry analysis of PBMCs and tissue lymphocyte subpopulations.

**Target**	**Specificity**	**Monoclonal/polyclonal**	**Primary antibody dilution**	**Reference**	**Secondary antibody**	**Secondary antibody dilution**
CD4	T helper lymphocytes	Monoclonal	1:400	MCA2213GA, BioRad	Polyclonal rabbit anti–mouse IgG-FITC, F0313, Dako	1:50
CD8	Cytotoxic T lymphocytes	Monoclonal	1:400	MCA2216GA, BioRad	Polyclonal rabbit anti–mouse IgG-FITC, F0313, Dako	1:50
WC1	γδ T lymphocytes	Monoclonal	1:200	MCA8586, BioRad	Polyclonal rabbit anti–mouse IgG-FITC, F0313, Dako	1:50
CD21	Naive B lymphocytes	Monoclonal	1:10	MCA1185, BioRad	Polyclonal rabbit anti–mouse IgG-FITC, F0313, Dako	1:50
CD20	Mature B lymphocytes	Polyclonal	1:100	9013-P, Thermo Scientific	Polyclonal goat anti–rabbit IgG-Alexa Fluor® 488, ab150077, Abcam	1:2,000
CD3	T lymphocytes, natural killer	Polyclonal	1:100	A0452, Dako	Polyclonal goat Anti- rabbit IgG-Alexa Fluor® 488, ab150077, Abcam	1:2,000

### Histopathological Examination

Fixed tissues for histopathological examination were conventionally processed for paraffin embedding and stained with hematoxylin–eosin and Ziehl–Neelsen technique for acid-fast bacilli detection (46).

Lesions consistent with *Map* infection were classified as *focal, multifocal a* and *multifocal b*, or *diffuse* forms according to the presence and location of granulomas and following the guidelines previously described for small ruminants (8, 46). Briefly, lesions were characterized as *focal* forms when granulomas were restricted to the lymphoid tissue of the Peyer patches; *multifocal* forms when the granulomas were located in the lamina propria adjacent to the lymphoid tissue (*multifocal a*) or not (*multifocal b*) and *diffuse* forms when granulomas spread to wide areas of the mucosa. Classification of each animal was based on its most severe granulomatous lesion. Following histopathological examination, the number of granulomas per tissue section was quantified in all tissue samples as described elsewhere (11, 46). Three tissue sections of each intestinal site (i.e., ileum—proximal, medium, and distal zones; jejunum—proximal, medium, and distal zones; ileocecal valve and jejunal Peyer patches—proximal, medium, and distal zones) and two sections from each lymph node (i.e., mesenteric, jejunal and ileocecal lymph nodes), were selected, and the mean number of granulomas per animal was recorded by the same observer (V.P.; diplomat of the European College of Veterinary Pathologists), distinguishing those granulomas located in the lymphoid tissue from those located in the associated lamina propria or in the mucosa not related to lymphoid tissue.

### Statistical Analysis

Normal distribution of the results from *Map*- and *Mbv*-specific antibodies, local and peripheral IFN-γ production, and proportion of peripheral and tissue lymphocyte subpopulations were assessed for normality using Shapiro–Wilk test. Data from IFN-γ production and antibody ELISA tests were logarithmically transformed. Then, flow cytometry and ELISA results from peripheral IFN-γ and antibody production were analyzed using generalized lineal model (GLM) procedure for evaluation of the main effects of vaccination, challenge, time, and its interactions. Subsequently, differences between vaccination groups for each time sampled were estimated using Tukey–Kramer correction for multiple comparisons. Similarly, the main effects of vaccination, tissue, and its interactions were estimated in the results of local IFN-γ production followed by the evaluation of differences between vaccination groups and tissues using Tukey–Kramer multiple-comparisons test. Besides, differences between groups in the number of goats with lesions were evaluated using χ^2^ and Fisher exact tests. In addition, after logarithmic transformation, differences in the granuloma counts between vaccination groups were calculated using the Student *t*-test. All statistical analyses were carried out using GraphPad Prism 6.0 software (San Diego, CA, USA) excluding GLMs that were performed using R software 3.5.3 (R Development Core Team, 2019). *P* < 0.05 was considered statistically significant.

## Results

### Peripheral and Local Cell-Mediated Immune Response (IFN-γ)

**Figure 2:**
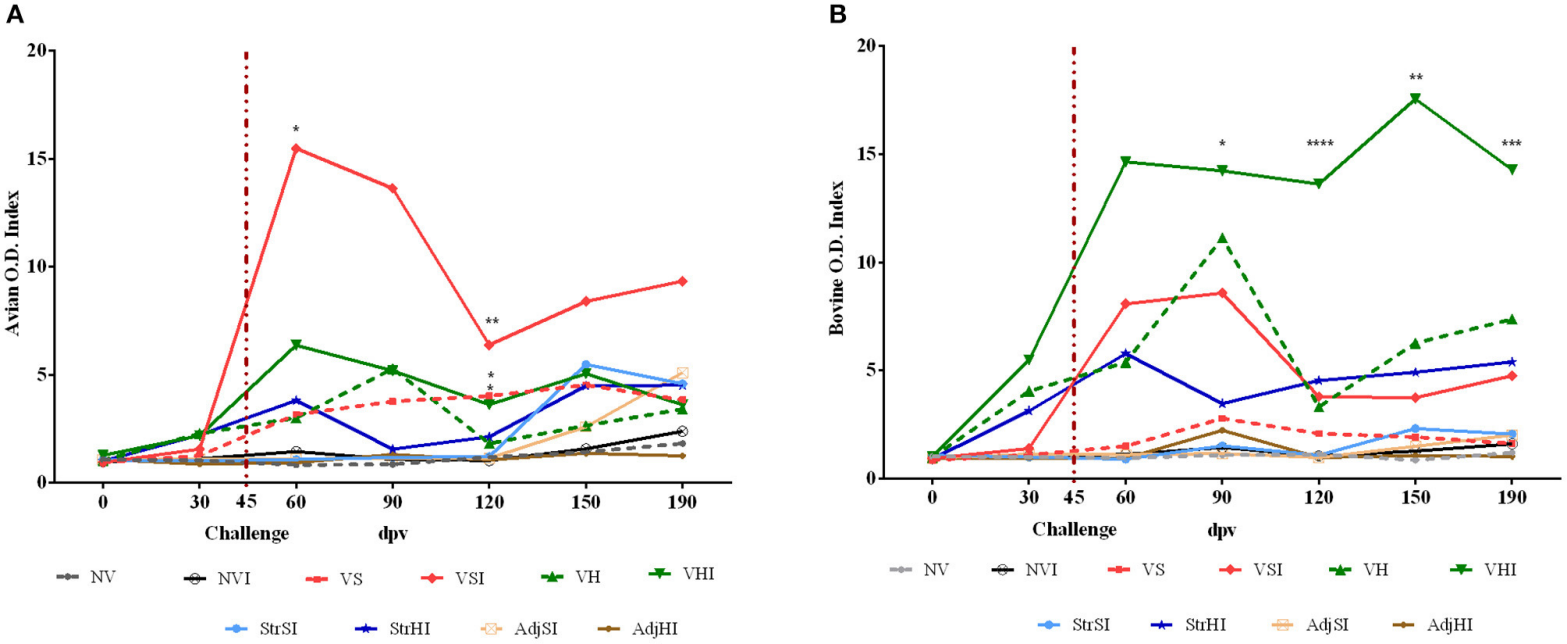
Kinetics of the IFN-γ production by whole blood stimulated with avian (PPDa) and bovine (PPDb) antigens. Results are expressed as avian **(A)** and bovine **(B)** O.D. index values of each vaccination group (*n* = 5) (NV, nonvaccinated and noninfected; NVI, nonvaccinated and infected; VS, Silirum® vaccinated and noninfected; VSI, Silirum® vaccinated and infected; VH, HIMB vaccinated and noninfected; VHI, HIMB vaccinated and infected; StrSI, *Map* 316F Silirum® strain immunized and infected; StrHI, *Mbv* 1403 HIMB strain immunized and infected; AdjSI, Montanide™ Silirum® adjuvant immunized and infected; AdjHI, Montanide™ HIMB adjuvant immunized and infected) and time sampled (dpv, days postvaccination). Vertical dotted red line represents the time of *Map* oral challenge (45 dpv). Significant differences determined by multiple comparisons were represented as *p < 0.05, **p < 0.01, ***p < 0.001, ****p < 0.0001.

Results from GLM showed that avian and bovine index values reached significant levels from 60 to 190 dpv in the VS, VSI, VH, and VHI groups (*p* < 0.001 and *p* < 0.01 respectively) (Figures 2A,B). In addition, Silirum® (VS and VSI) and HIMB vaccine (VH and VHI) immunization exerted a considerable effect on avian index values regardless of the infection status (*p* < 0.01 and *p* < 0.001, respectively) (Figure 2A), but only the VHI, VH, and StrHI groups showed a significant effect on bovine index values (*p* < 0.01) (Figure 2B). Furthermore, oral challenge significantly impacted on the avian index levels of the VSI, VHI (*p* < 0.001), and NVI (*p* < 0.05) groups and on the bovine index values of the VSI, VHI (*p* < 0.01), and StrHI (*p* < 0.05) groups.

Multiple-comparisons analysis showed that the VSI group had higher avian index values than NV, NVI, StrSI, AdjSI, and AdjHI at 60 and 120 dpv, whereas the VS group showed these differences only at 120 dpv (Figure 2A). In contrast, these groups did not show any significant differences in bovine index levels (Figure 2B). Furthermore, no differences were observed between VS and VSI either in avian or bovine index values at any time point. On the other hand, the VHI group showed greater avian index values than NVI, NV, StrSI, AdjSI, and AdjHI at 120 dpv (Figure 2A). In addition, bovine index values of this group were significantly higher than NV, NVI, StrSI, AdjSI, and AdjHI at 90, 120, 150, and 190 dpv and greater than VH at 120 dpv (Figure 2B).

**Figure 3:**
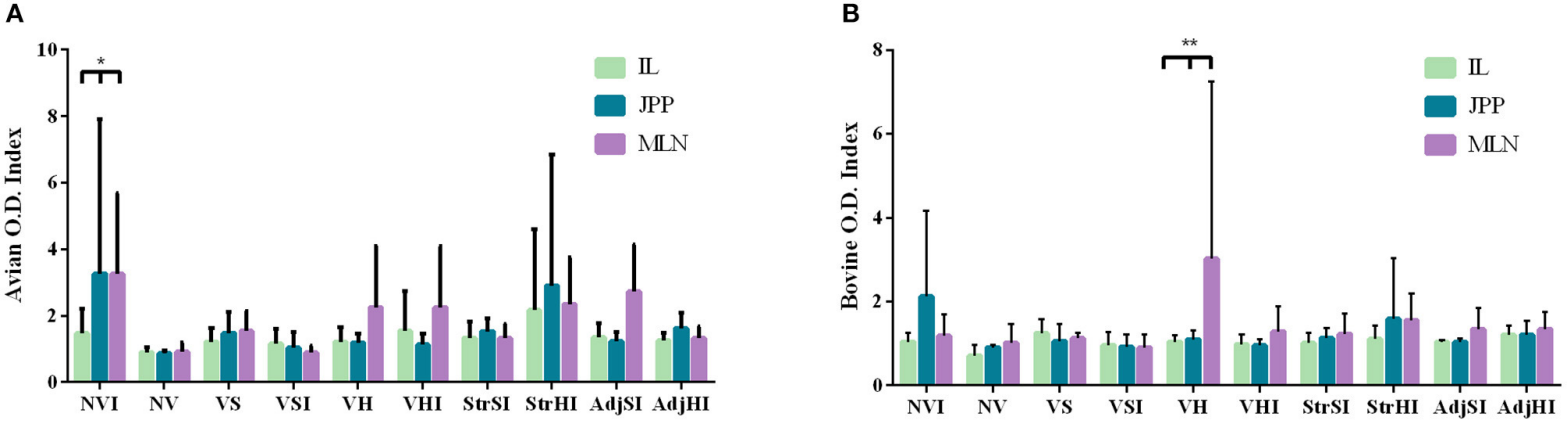
IFN-γ production by leukocytes from ileum (IL), jejunal Peyer patches (JPP), and mesenteric lymph node (MLN) stimulated with avian (PPDa) and bovine (PPDb) antigens. Results are expressed as avian **(A)** and bovine **(B)** O.D. index values of each vaccination group (*n* = 5) (NV, nonvaccinated and noninfected; NVI, nonvaccinated and infected; VS, Silirum® vaccinated and noninfected; VSI, Silirum® vaccinated and infected; VH, HIMB vaccinated and noninfected; VHI, HIMB vaccinated and infected; StrSI, *Map* 316F Silirum® strain immunized and infected; StrHI, *Mbv* 1403 HIMB strain immunized and infected; AdjSI, Montanide™ Silirum® immunized and infected; AdjHI, Montanide™ HIMB adjuvant immunized and infected). Bars and vertical lines represent mean values and standard deviations, respectively. Significant differences determined by multiple comparisons were represented as *p < 0.05, **p < 0.01, ***p < 0.001, ****p < 0.0001.

Local IFN-γ production in mononuclear leukocytes purified from tissues and stimulated with PPDa and PPDb was lower than that observed in PBMCs. Furthermore, avian index values were more heterogeneous than bovine index levels on account of the higher individual variability observed in the former (Figures 3A,B), specifically at the NVI, VH, VHI, StrHI, and AdjSI groups (Figure 3A). Peyer patches and mesenteric lymph node from NVI group showed the highest avian index values followed by the ileum, Peyer patches, and mesenteric lymph node from the StrHI group (Figure 3A). However, statistical analysis showed no significant effect of vaccination or tissue location on the avian or bovine index values (*p* > 0.05). In addition, no significant differences were observed between the vaccinated groups in the multiple-comparisons analysis either (*p* < 0.05). Meanwhile, significant differences were observed in the avian index values within the NVI group, where IFN-γ production was higher in Peyer patches and mesenteric lymph node than in the ileum (*p* < 0.05) (Figure 3A). Besides, despite the high variability within groups observed in the bovine index values, the values of mesenteric lymph node from the VH group were considerably higher than those of the ileum and Peyer patches (*p* < 0.01) (Figure 3B).

### Peripheral Humoral Response

**Figure 4:**
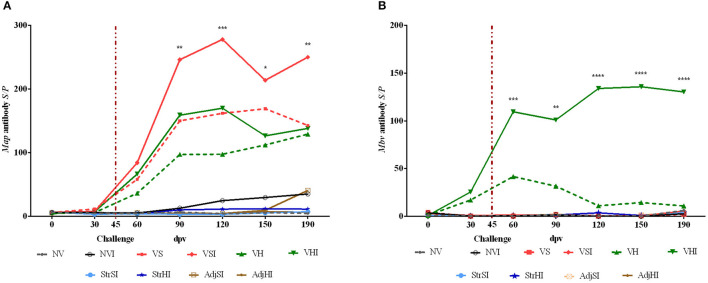
Kinetics of peripheral blood serum antibody levels. Results are expressed as *S/P* ratio of *Map* **(A)** and *Mbv* **(B)** specific antibodies of each vaccination group (*n* = 5) (NV, nonvaccinated and noninfected; NVI, nonvaccinated and infected; VS, Silirum® vaccinated and noninfected; VSI, Silirum® vaccinated and infected; VH, HIMB vaccinated and noninfected; VHI, HIMB vaccinated and infected; StrSI, *Map* 316F Silirum® strain immunized and infected; StrHI, *Mbv* 1403 HIMB strain immunized and infected; AdjSI, Montanide™ Silirum® immunized and infected; AdjHI, Montanide™ HIMB adjuvant immunized and infected) and time sampled (dpv, days postvaccination). Vertical dotted red line represents the time of *Map* oral challenge (45 dpv). Significant differences determined by multiple comparisons were represented as *p < 0.05, **p < 0.01, ***p < 0.001, ****p < 0.0001.

Results from GLM estimated that both *Map*- and *Mbv*-specific antibody production reached significant levels from 60 to 190 dpv (*p* < 0.001 and *p* < 0.0001, respectively) in Silirum® and HIMB-vaccinated groups (Figures 4A,B). In addition, oral challenge boosted *Map*-specific antibody production in the vaccinated groups VSI, VHI (*p* < 0.001), and NVI (*p* < 0.05) (Figure 4A) and *Mbv*-specific antibody production in VHI (*p* < 0.001) (Figure 4B). Besides, multiple-comparisons analysis showed that only VSI had significantly higher *Map*-specific *S/P* values in comparison with the groups immunized with adjuvants, inactivated bacteria, and nonvaccinated at 90, 120, 150, and 190 dpv, whereas no significant differences were observed in VHI (Figure 4A). Additionally, no significant differences were observed in the *Map* antibody levels either between Silirum® and HIMB vaccination groups or between infected and noninfected vaccinated groups.

*Mbv*-specific *S/P* values were significantly higher when comparing the VHI group with the VS, VSI, StrSI, StrHI, AdjSI, and AdjHI groups at 60, 90, 120, 150, and 190 dpv (Figure 4B). However, significant differences between VHI and VH were observed only at 120, 150, and 190 dpv (Figure 4B).

### Quantitative PCR and Bacteriology of Tissues and Feces

*Map* detection was confirmed by qPCR (*n* = 4) and bacteriological culture (*n* = 3) within infected goats (*n* = 35). Animals positive to qPCR corresponded to (i) two goats from StrHI (mesenteric lymph node and the Peyer patches, respectively), (ii) one from VHI (Peyer patches), and (iii) one from AdjSI (Peyer patches). Besides, animals positive to bacteriological culture corresponded to one goat each from NVI, VHI (ileum, Peyer patches, and mesenteric lymph node positives), and VSI (ileum and mesenteric lymph node positives). Only the Peyer patches from the goat from VHI showed positive results for both detection techniques. All tissue samples from noninfected groups were negative to IS*900* sequence amplification by qPCR or bacteriological culture. Bacteriological culture of *Map* was negative in every fecal sample analyzed.

### Relative Peripheral Blood and Tissue Lymphocyte Subsets

The mean and standard deviations of the relative proportions of three T lymphocytes (CD4^+^, CD8^+^, and CD3^+^), one γδ T lymphocyte, and two B lymphocyte (CD21^+^ and CD20^+^) surface markers were estimated by flow cytometry.

Along all groups, the highest relative proportion of positive cells in PBMCs isolated from whole blood corresponded to γδ T lymphocytes (30.46% ± 3.55%), whereas the lowest proportion was observed in CD20^+^ B lymphocytes (5.96 ± 1.48%) throughout the study (Supplementary Table 1). However, despite statistical analysis showing significant oscillations (*p* < 0.001) of the relative proportions of CD4^+^, CD8^+^, WC1^+^, CD21^+^, CD3^+^, and CD20^+^ lymphocytes at different samplings, no significant differences were found between groups (*p* > 0.05).

Flow cytometry carried out in cells purified from tissue samples showed that the highest relative proportion levels corresponded to CD21^+^ B lymphocytes in the ileum (39.47 ± 4.71%), Peyer patches (26.81 ± 3.59%), and mesenteric lymph node (44.90 ± 3.52%), whereas γδ T lymphocytes were the scarcest cell population in these samples (0.99 ± 0.24%, 1.69 ± 0.33%, and 0.92 ± 0.26%, respectively). Statistical analysis showed a significant influence of vaccination and immunization with inactivated bacteria and adjuvants on the relative proportion of tissue lymphocyte subsets (*p* < 0.05). However, results from comparisons between groups did not show a clear pattern between the relative proportion of lymphocyte subpopulations and the different groups likely on account of the individual variability observed within groups. Mean, standard deviation, and results of multiple comparisons of lymphocyte relative subpopulations in ileum, Peyer patches, and mesenteric lymph node are summarized in Supplementary Tables 2–4.

### Pathological Findings

**Figure 5:**
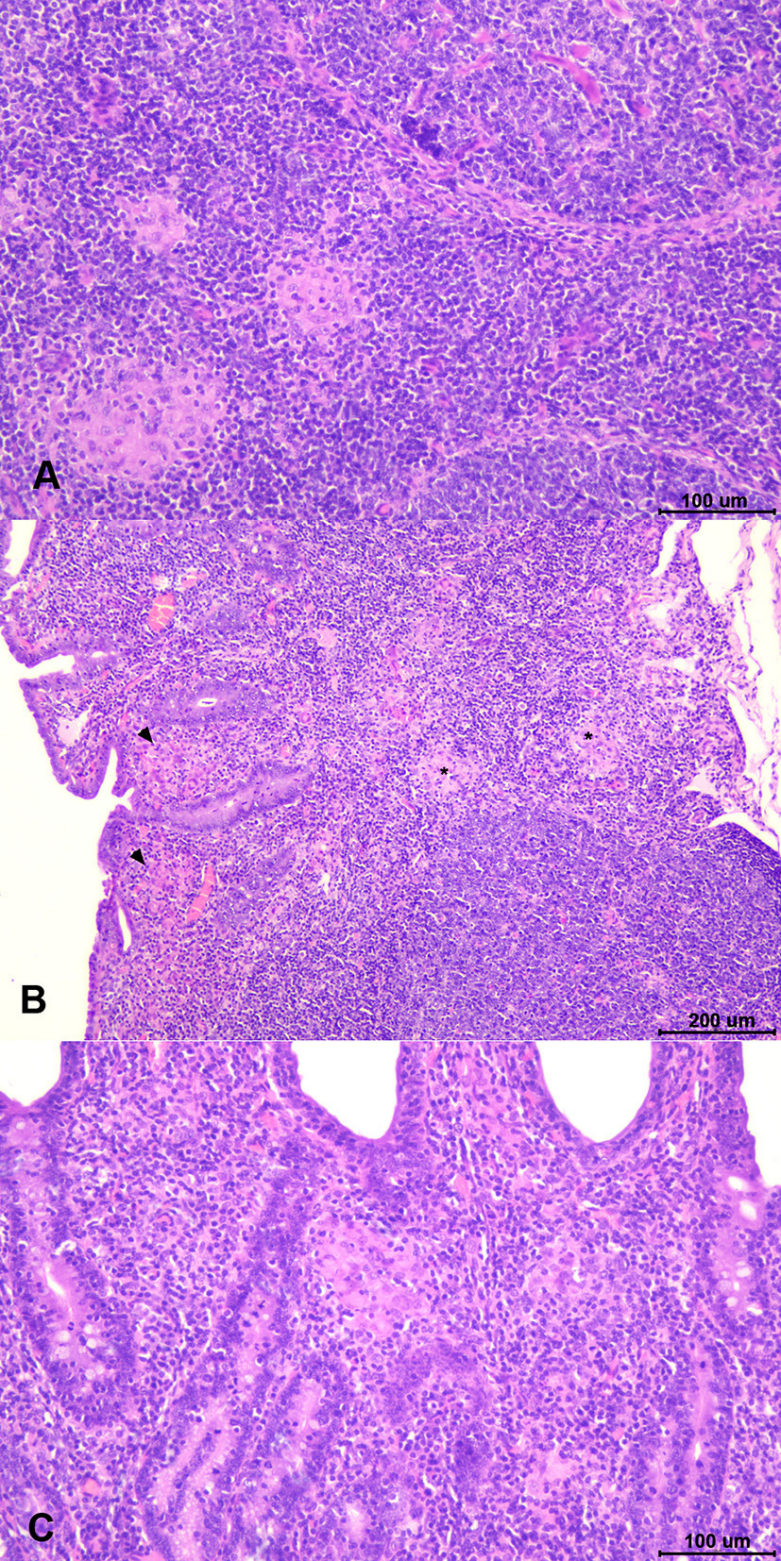
Types of paratuberculosis granulomatous lesions found in the study. **(A)** *Focal* lesion: well-demarcated granulomas composed of small groups of macrophages located in the interfollicular area of the Peyer patches. **(B)** *Multifocal a* lesion: small granulomas located in the interfollicular area of the lymphoid tissue (asterisk) and in the associated lamina propria (arrowheads). **(C)** *Multifocal b* lesion: granulomas located in the lamina propria in area devoid of lymphoid tissue.

Gross lesions compatible with paratuberculosis were not found in any animal. Microscopic granulomatous lesions characteristic of *Map* infection were detected in all infected groups, with differences in the severity and distribution. The number of goats per group with lesions according to the tissue and the number of goats per group classified in terms of severity of the lesions are shown in Table 2. Goats with small and well-defined granulomas composed of macrophages, with an abundant pale cytoplasm and a large nucleus, escorted by a few lymphocytes located exclusively in the interfollicular area of the intestinal lymphoid tissue were classified as *focal* (*n* = 5) (Figure 5A). Besides, goats with well-defined granulomas in the interfollicular area of the Peyer patches and also in the lamina propria closely associated with the intestinal lymphoid tissue were categorized as *multifocal a* (*n* = 5) (Figure 5B). Finally, the presence of granulomatous lesions not only in the Peyer patches and related lamina propria but also in areas of the mucosa without any association with the lymphoid tissue was considered as *multifocal b* (*n* = 13) (Figure 5C). No *diffuse* lesions were noticed in the tissues of any goat (*n* = 0). VSI and VHI groups were the less affected with only one animal from each group developing granulomatous lesions, showing significant differences in comparison to NVI in which lesions were present in all animals (*p* < 0.05). In addition, lesions from the VSI goat were classified as *focal*, whereas the goat from VHI group showed more severe lesions, categorized as *multifocal b* as all goats from the NVI. Besides, most of the goats immunized with strains (StrSI and StrHI) developed *multifocal b* forms, except for one animal from StrHI with a *focal* lesion. In contrast, among the animals immunized with adjuvants (AdjSI and AdjHI), only one animal from the AdjSI showed *multifocal b* lesions, whereas *focal* and *multifocal a* lesions were found in the remaining goats. No significant differences were detected in the number of goats with granulomatous lesions in groups immunized with inactivated bacteria or adjuvants (*p* > 0.05). Granulomatous lesions were localized mainly in the jejunal Peyer patches followed by distal ileum, lymph nodes, ileocecal valve, and jejunum, although lesions observed in the lymph nodes were always associated with the presence of granulomatous lesions in the intestine (Table 3). No microscopic lesions were observed in the groups that remained as noninfected. No acid-fast bacilli were detected in the lesions from any of the infected goats.

**Table 2:** Number of animals from each group with microscopic lesions consistent with paratuberculosis according to the examined tissue and its severity (*focal, multifocal a, multifocal b*, and *diffuse*).

**Group (*n* = 5)**	**Tissues**							**Severity**				
	**IL**	**JJ**	**JPP**	**ICV**	**MLN**	**JLN**	**ICLN**	***Focal***	***Multifocal a***	***Multifocal b***	***Diffuse***	**Total**
NVI	3/5	2/5	5/5	3/5	1/5	1/5	—	—	—	5/5	—	5/5
VSI	1/5	—	1/5	—	—	—	1/5	1/5	—	—	—	**1/5** ^**a**^ ^*^
VHI	1/5	—	1/5	—	—	—	—	—	—	1/5	—	**1/5** ^**a**^ ^*^
StrSI	3/5	2/5	3/5	1/5	3/5	1/5	1/5	—	—	3/5	—	3/5
StrHI	1/5	—	4/5	1/5	1/5	1/5	—	1/5	—	3/5	—	4/5
AdjSI	1/5	—	3/5	1/5	—	—	—	—	2/5	1/5	—	3/5
AdjHI	1/5	—	3/5	1/5	1/5	—	—	3/5	1/5	—	—	4/5

*IL, Ileum; JJ, jejunum; JPP, jejunal Peyer patches; ICV, ileocecal valve; MLN, mesenteric lymph node; JLN, jejunal lymph node; ICLN, ileocecal lymph node; NVI, Nonvaccinated and infected; VSI, Silirum® vaccinated and infected; VHI, HIMB vaccinated and infected; StrSI, Map 316F Silirum® strain immunized and infected; StrHI, Mbv 1403 HIMB strain immunized and infected; AdjSI, Montanide™ Silirum® adjuvant immunized and infected; AdjHI, Montanide™ HIMB adjuvant immunized and infected. Significant differences detected by χ^2^ and Fisher exact test are expressed as*

* *p < 0.05*,

**p < 0.01, ***p < 0.001, and ****p < 0.0001.

—*No lesion*.

a *Significantly different from NVI*.

**Table 3:** Results of granuloma count. Number of granulomas according to their location for each group and total number and percentage (%) of granulomas per group.

**Group (*n* = 5)**	**IL**	**JJ**	**JPP**	**ICV**	**MLN**	**JLN**	**ICLN**	**Total number**
NVI	35	6	690	33	12	22	0	798 (47.10%)
VSI	2	0	12	0	0	0	9	23 (1.36%)
VHI	2	0	30	0	0	0	0	31 (1.83%)
StrSI	39	6	186	7	29	2	19	288 (17.00%)
StrHI	3	0	293	8	3	1	0	308 (18.18%)
AdjSI	4	0	92	4	0	0	0	100 (5.90%)
AdjHI	21	0	83	3	39	0	0	146 (8.63%)
Total	106	12	1,386	55	83	25	28	1,694 (100%)

*IL, Ileum; JJ, jejunum; JPP, jejunal Peyer patches; ICV, ileocecal valve; MLN, mesenteric lymph node; JLN, jejunal lymph node; ICLN, ileocecal lymph node; NVI, Nonvaccinated and infected; VSI, Silirum® vaccinated and infected; VHI, HIMB vaccinated and infected; StrSI, Map 316F Silirum®strain immunized and infected; StrHI, Mbv 1403 HIMB strain immunized and infected; AdjSI, Montanide™ Silirum® adjuvant immunized and infected; AdjHI, Montanide® HIMB adjuvant immunized and infected*.

**Figure 6:**
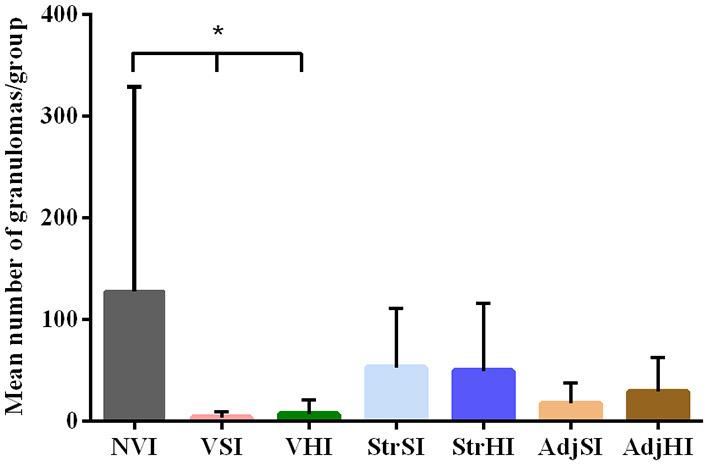
Mean granuloma count from the tissues of vaccinated and infected groups. Data are expressed as mean number of granulomas per group, and standard deviations in each group (*n* = 5) and significant differences were represented as *p < 0.05, **p < 0.01, ***p < 0.001, ****p < 0.0001.

The total number of granulomas per group was summarized in Table 3. The NVI group showed the highest number, whereas the lowest corresponded to the VSI and VHI groups. In addition, the number of granulomas observed in the StrSI and StrHI groups was higher than those observed in AdjSI and AdjHI. Significant differences in the mean number of granulomas per group (Figure 6) were observed between NVI (159.60 ± 271.90) and VSI (4.60 ± 10.29) and VHI (6.20 ± 13.86) groups (*p* < 0.05). However, no significant differences were found in the StrSI (57.60 ± 61.54), StrHI (61.40 ± 83.22), AdjSI (20.00 ± 26.82), and AdjHI (29.20 ± 33.86) groups (*p* > 0.05).

## Discussion

Paratuberculosis vaccination studies conducted in ruminants have demonstrated worthwhile results in the reduction of the number of clinically affected animals and the prevalence of the disease (16, 23). Nevertheless, the inability of the protective immune response elicited by vaccination to completely prevent the infection and its progression and spread is alienating from the term “ideal vaccine” (47, 48). This handicap is partly due to the lack of knowledge about the peripheral and local mechanisms related to the success or failure of the protection against mycobacteria. Here, this work analyzes the local and peripheral immune response elicited by homologous (Silirum®) and heterologous (HIMB vaccine) vaccination and the immunization with their components separately and its relation to the protection degree estimated by the evaluation of the microscopic granulomatous lesions and *Map* presence in the tissues.

Despite the identification of distinctive paratuberculosis granulomatous lesions in all groups of infected goats, regardless of its vaccination or immunization status, the variation in their severity mirrors the observations from field and experimental studies, as lesions observed in paratuberculosis-vaccinated goats were mild (*focal*) and scarce, whereas those in nonvaccinated and infected goats were more severe (*multifocal b*), proving the high protective effect of vaccination (16, 22, 41). No *diffuse* lesions were noticed, possible due to the short incubation period of this work (4–5 months), in line with other studies in which *multifocal b* lesions were observed up to 7 months postinfection, whereas *diffuse*-type lesions were not reported until 12 months (46, 49). Despite this fact, the results obtained here agree with other experimental studies in which lesions observed in vaccinated animals were limited to regressive forms (*focal*), contained in the interfollicular area of the intestinal lymphoid tissue, avoiding their dissemination through the intestine but without preventing infection as it was confirmed by the detection of *Map* in tissues from a VSI goat (41, 50, 51).

To date, *in vivo* vaccination efficacy studies have been mainly focused on the early and sustained induction of peripheral IFN-γ (20, 21, 52) as a release of this cytokine has been associated with the activation of antimycobacterial mechanisms and the prevention of intracellular bacterial growth (53, 54). Certainly, IFN-γ production dynamics observed in Silirum®-vaccinated groups (i.e., VS and VSI) was consistent with other studies conducted using goat kids vaccinated with heat-killed vaccines (52, 55). In addition to cell-mediated immune response, Silirum®-vaccinated animals also showed increased *Map* antibody levels. The role of humoral response has been underestimated as it has been considered ineffective against intracellular pathogens (56, 57). However, an emerging number of studies performed under experimental conditions have suggested the potential positive effect of antibodies in the control of paratuberculosis, even in relation to vaccination (58–60). This is supported by the initial strong humoral response observed in paratuberculosis-vaccinated sheep and the ability of these animals to modulate that response in later stages to prevent the progression of infection (60). Both cellular and humoral responses were significantly enhanced in this study after experimental challenge with *Map*. These results mimic the increase in IFN-γ observed in paratuberculosis-vaccinated and naturally exposed goats in field conditions (61), although the significant increase in the antibody production after *Map* oral challenge here observed contrasts with previous studies where experimental oral infection in vaccinated sheep did not exhibit differences with nonvaccinated animals or even lead to a decrease in the antibody production (22, 60), although differences in the adjuvant of the vaccine used in that study (i.e., Gudair®) or the species (i.e., sheep) may explain these discrepancies. Nevertheless, the heightened cellular and humoral responses here observed did not prevent the appearance of granulomatous lesions in VSI group. Thus, despite that vaccination might be limiting the progression and severity of the lesions, as only one VSI goat developed a low number of *focal* granulomas, peripheral cellular and humoral response did not allow predicting the presence of lesions or infection in vaccinated animals.

In addition, changes in the relative proportion of lymphocyte subpopulations have also been connected to mycobacterial infections (62–64). In this sense, cows with clinical disease have shown lower proportions of CD4^+^, CD8^+^, and γδ T lymphocytes than subclinical cows in freshly isolated PBMCs (65). Furthermore, in another study, higher percentages of γδ T and B lymphocytes and a decline in CD4^+^ were detected in tissues of infected sheep during early *Map* experimental infection (10). Regarding the effect of vaccination, it has been reported that infection of Gudair®-vaccinated sheep with *Map* leads to a decrease in the proportions of peripheral CD4^+^ and B lymphocytes at 13 days postchallenge assessed in *in vitro Map*-stimulated PBMCs, but this was not evaluated in nonstimulated and freshly isolated cells (22). On the contrary, no changes in the relative proportion of either peripheral T lymphocytes (CD4^+^, CD8^+^, γδ, and CD3^+^) or B lymphocytes (CD20^+^ and CD21^+^) were detected in freshly isolated PBMCs between any group in this study. In addition, differences in the tissue relative proportion of lymphocyte subpopulations were estimated, but no clear relationship between these results and vaccination/immunization and/or infection was observed, likely due to the high individual variability within tested groups. Similarly, no relation between local production of IFN-γ and development of lesions either in VSI or other infected groups was observed. In this sense, only the Peyer patches and mesenteric lymph node from NVI group produced a slightly higher IFN-γ production. This result is consistent with the greater number of granulomas detected in this group as an increased number of granulomatous lesions have been related to a higher number of IFN-γ-immunolabeled cells (6) and are likely that lesions present in the remaining infected groups might not be sufficient to generate a substantial local cell-mediated immune response in response to mycobacterial antigens. Therefore, neither peripheral cellular and humoral immune responses nor evaluation of the relative proportion of lymphocyte subpopulations in whole blood and tissues nor local production of IFN-γ was able to predict those animals that remain unprotected early after infection. Besides, vaccination has demonstrated to reduce the number of excreted bacteria both in field and experimental conditions (17, 21, 37, 66). In this study, *Map* was not isolated in the feces of any goat. Heightened *Map* shedding has been detected in relation to the development of severe lesions and clinical signs in naturally and experimentally infected animals (67, 68), although it could be intermittently detected in animals with subclinical paratuberculosis (53, 69). However, feces were collected only at sacrifice (145 days postinfection), so the presence of *Map* could have been underestimated because of the short postchallenge time and the limited time sampled. Moreover, it has been observed that fecal shedding is frequently associated with animals with a high number of culture-positive tissues and severe lesions (70). In addition, the low number of *Map*-positive tissues detected either by bacteriological culture or qPCR (7 of 35 infected goats) was not surprising as in several previous works, either in natural or experimental cases (46, 71), it has been shown that the bacterial load in animals with lesions similar to those found in this study was absent or very low.

Interestingly, only one goat from the VHI group developed paratuberculosis granulomatous lesions, albeit classified as *multifocal b*, showing the effectiveness of heterologous protection that vaccination for tuberculosis confers against *Map* infection. Similar to homologous vaccination, HIMB-vaccinated groups (i.e., VH and VHI) showed a significant IFN-γ and antibody production as previously described in goats (66, 72), even when blood was stimulated with PPDa. In addition, boost in the IFN-γ production was also noticed in goats vaccinated subcutaneously with HIMB vaccine and endobronchially challenged with a closely related mycobacteria (*M. caprae*) under experimental conditions (66), proving again that the elevation of these responses does not ensure the success of vaccination as in the VHI goat, granulomatous lesions were more severe (*multifocal b*) than those from the VSI group (*focal*). Cross-protection effect related to vaccination has been previously reported as a reduction of pulmonary lesions was noticed in paratuberculosis-vaccinated goats (32) or calves (33) endobronchially infected with *M. caprae* or *Mbv*, respectively. Besides, despite this beneficial effect, cross-reaction against standard mycobacterial antigens was noticed in IFN-γ release assay and *Map*-specific antibody determination and entailed a relevant downside for the differentiation between infected and vaccinated animals (DIVA) (31). This cross-reaction has been previously described in response to PPDa and PPDb mycobacterial antigens, although usually this production is biased toward the antigen related to the homologous stimulation (73–75). It is remarkable that specific antibodies against *Mbv* were detected only in the VHI group and, for a short while, in the VH group, whereas specific antibodies against *Map* were readily found not only in the VSI group, but also in the VS, VH, and VHI groups. Recent advances in the evaluation of recombinant proteins for *Mbv* serological tests, as the one used here (MBP83), have made it possible to minimize the cross-reaction induced by paratuberculosis vaccination on tuberculosis diagnosis (38, 76). Therefore, the development and use/implementation of more specific antigens (DIVA reagents) for both IFN-γ assay and antibody detection are necessary in order to improve the immunological diagnosis of paratuberculosis and tuberculosis and to overcome the interferences produced by vaccination (31).

Addressing the effect of Silirum® and HIMB inactivated strains individually, it was observed that goats from StrSI and StrHI showed a higher number of granulomatous lesions affecting a greater number of animals than those of the VSI or VHI groups. In addition, despite the fact that the number of granulomas was lower than that in the NVI goats, these were also classified as *multifocal b*, proving the progression of infection in these groups. Regarding peripheral immune responses, only the StrHI group showed high peripheral IFN-γ levels in response to both mycobacterial antigens but not the production of peripheral antibodies, which could suggest the ability of this strain to stimulate only the peripheral cellular immune response. In this sense, in a study conducted in cattle, oral administration of inactivated *Mbv* 1403 strain suspended in PBS did not show an increased IFN-γ production in response to bovine antigen, whereas the parenteral immunization with this strain homogenized in Montanide™ ISA 50 V 2 adjuvant (HIMB vaccine) showed an elevation of the IFN-γ production from 2 weeks postimmunization, although the effect of the inactivated bacteria administered parenterally was not evaluated in absence of the adjuvant (77). On the contrary, no evident peripheral cellular or humoral immune responses were detected in the StrSI group, although IFN-γ rose at 150 dpv. In this sense, it is feasible that this late increase was the effect of *Map* infection rather than the immunization itself, as the animals with the highest cell-mediated peripheral immune response corresponded to those with the most numerous granulomatous lesions (data not shown). Thus, this limited peripheral immune response elicited by inactivated bacteria might be a consequence of their weak immunogenicity in absence of adjuvants (78, 79).

Besides, the immunization with adjuvants (i.e., AdjSI and AdjHI) in the absence of bacterial antigens resulted in a lower number of granulomatous lesions than in the StrSI, StrHI, or even NVI group. Furthermore, these lesions were mainly classified as *focal* and *multifocal a*, with only one animal from the AdjSI developing *multifocal b* lesions. In this sense, no significant peripheral cellular or humoral immune responses were detected in adjuvant immunized groups. This is in agreement with a previous study where no specific peripheral immune response was recorded in Montanide™ ISA 50 V 2 adjuvant immunized calves (80). However, only AdjSI showed a later increase in peripheral IFN-γ response (150 dpv) and *Map*-specific antibodies (190 dpv) possibly associated with the presence of *multifocal a* and *b* lesions in contrast to AdjHI that showed mainly *focal* lesions similar to the VSI group, although the main effect of adjuvants could not be assessed because of the lack of noninfected adjuvant immunized groups. This is similar to the later increase observed in StrSI, StrHI, and NVI, in which also *multifocal b* lesions were detected. Therefore, it is possible that the greater number and extension of the lesions in these groups were correlated with a higher response from sensitized lymphocytes that might have migrated from the intestine to blood, as it was previously hypothesized (6).

The main role of mineral oil adjuvants in vaccination is to increase the immune response through the sustained release of the antigen at the site of injection, ensuring the constant stimulation of the immune response (78). Montanide™ adjuvants, as used here, provoke a liposome that protects the inactivated antigen from degradation, driving it to draining lymph nodes through the recruitment of antigen-presenting cells and lymphocytes (79). However, differences in the immune response profiles depending on the type of Montanide™ adjuvant have been reported (25, 81, 82). For instance, the antigen-specific IFN-γ and *Map*-specific antibody responses elicited by the vaccination of sheep with heat-killed *Map* 316F strain combined with Montanide™ ISA 50 V 2 were lower than those provoked by vaccination with Gudair® commercial vaccine (Montanide™ ISA 103 and 80) (25). These differences could be related to the composition of adjuvants (type of fatty acids or emulsifier) and the intensity of the inflammatory response at the site of injection, however, and despite their importance in vaccine formulation, their primary interaction with the host immune response is not yet fully understood, as their effect is often studied in combination with antigens (26). For that reason, despite the absence of statistical differences on account of the high individual variability, it is striking that the limited number and severity of granulomatous lesions in AdjSI and AdjHI groups without the release of an antigen were quite similar to those observed in the VSI and VHI groups. In a study conducted in calves, only two of six calves immunized with Montanide™ ISA 50 V 2 showed *Mbv* positive culture in the right prescapular lymph nodes after intranodular *Mbv* challenge (80), which suggest that the adjuvant might limit, in some extent, the spread of the infection. It is known that application of water-in-oil adjuvants showed a clear stimulatory effect over the host immune response prior to immunization with an antigen (27). However, in absence of a clear specific peripheral or local immune responses, it is tempting to hypothesize that this protective immune response could be mediated by nonspecific mechanisms that might help the host to control the mycobacterial infection. This mechanism is unknown, but other immune cells such as antigen-presenting cells (i.e., macrophages, dendritic cells) could be mediating in this response as they are recruited to the inoculation site (26) and possibly are being activated due to the inflammatory response. In fact, it has been hypothesized that these cells may be involved in the development of a “trained” immune response characterized by their heightened and nonspecific response after reinfection independently of the memory adaptive immune response (83).

Taken together, this study has evaluated the immunological profiles of two heat-inactivated vaccines against paratuberculosis and tuberculosis before and after *Map* challenge. Our results highlighted the fact that predictors such as peripheral and local IFN-γ response, peripheral antibody production, and peripheral and local evaluation of lymphocyte subpopulations are unable to assess the outcome of vaccination being only effectively evaluated by the assessment of microscopic lesions that has proven to be an effective method for estimating the infection status (9, 11, 46). However, both homologous vaccination and heterologous vaccination were able to confer a high protective immune response against *Map* infection, limiting the progression of granulomatous lesions and additionally proving the potential benefit of tuberculosis vaccination on the paratuberculosis control. In this sense, more studies focused on the reduction of interferences in the serological tests used for paratuberculosis and tuberculosis diagnosis possibly targeted on alternative vaccination routes or different adjuvant used are needed. Besides, an interesting effect in the reduction of the number and severity of paratuberculosis granulomatous lesions was observed in groups immunized with high refined mineral oil adjuvants. Therefore, more studies are needed to evaluate the effect of each vaccine components that could be involved in the protection against mycobacteria.

## Data Availability Statement

The raw data supporting the conclusions of this article will be made available by the authors, without undue reservation.

## Ethics Statement

The animal study was reviewed and approved by Subcommittee on Animal Experiments and Welfare of the University of León (ULE) (OEBA-ULE-016-2017).

## Author Contributions

VP, JB, DG-E, and NA-V designed and conducted the experiment. JE assisted in the statistical analysis. VP, JB, DG-E, NA-V, RV, and MF were involved in the necropsies as well as in blood and tissue samples collection and analysis. *Map* inoculum and HIMB vaccine preparation were carried out by NE and IS that, together with NA-V, participated in the bacteriological analysis. The manuscript was written by VP, JB, DG-E, and NA-V. The final submitted version was read and approved by all the authors.

## Funding

This work was financially supported by the Spanish Ministry of Science and Innovation (projects AGL2015-66540-C2-1-R and RTI2018-099496-B-I00), Junta de Castilla y León (project LE259P18), and National Institute for Agronomic Research (project RTA 2017-00089-00-00). NA-V was the recipient of a predoctoral contract (BES-2016-076513) from the Spanish Ministry of Science and Innovation and DG-E and JE of a postdoctoral contract from the Ministry of Science and Innovation (grants nos. FJCI-2017-32020 and FJC2019-042422-I respectively).

## Conflict of Interest

The authors declare that the research was conducted in the absence of any commercial or financial relationships that could be construed as a potential conflict of interest.

## Publisher's Note

All claims expressed in this article are solely those of the authors and do not necessarily represent those of their affiliated organizations, or those of the publisher, the editors and the reviewers. Any product that may be evaluated in this article, or claim that may be made by its manufacturer, is not guaranteed or endorsed by the publisher.

## References

[1] Rathnaiah G Zinniel DK Bannantine JP Stabel JR Gröhn YT Collins MT. Pathogenesis, molecular genetics, and genomics of *Mycobacterium avium* subsp. paratuberculosis, the etiologic agent of Johne's disease. Front Vet Sci. (2017) 4:187. 10.3389/fvets.2017.00187 29164142 PMC5681481

[2] Garcia AB Shalloo L. Invited review: the economic impact and control of paratuberculosis in cattle. J Dairy Sci. (2015) 98:5019–39. 10.3168/jds.2014-9241 26074241

[3] Rasmussen P Barkema HW Mason S Beaulieu E Hall DC. Economic losses due to Johne's disease (paratuberculosis) in dairy cattle. J Dairy Sci. (2021) 104:3123–43. 10.3168/jds.2020-19381 33455766

[4] Clarke CJ. The pathology and pathogenesis of paratuberculosis in ruminants and other species. J Comp Path. (1997) 116:217–61. 10.1016/S0021-9975(97)80001-1 9147244

[5] Stabel JR. Transitions in immune responses to *Mycobacterium paratuberculosis*. Vet Microbiol. (2000) 77:465–73. 10.1016/S0378-1135(00)00331-X 11118731

[6] Fernández M Fuertes M Elguezabal N Castaño P Royo M Ferreras MC. Immunohistochemical expression of interferon-γ in different types of granulomatous lesions associated with bovine paratuberculosis. Comp Immunol Microbiol Infect Dis. (2017) 51:1–8. 10.1016/j.cimid.2017.01.002 28504089

[7] Pérez V García Marín JF Badiola JJ. Description and classification of different types of lesion associated with natural paratuberculosis infection in sheep. J Comp Path. (1996) 114:107–22. 10.1016/S0021-9975(96)80001-6 8920212

[8] Corpa JM Garrido J García Marín JF Pérez V. Classification of lesions observed in natural cases of paratuberculosis in goats. J Comp Path. (2000) 122:255–65. 10.1053/jcpa.1999.0368 10805979

[9] Begg DJ. O‘Brien R, Mackintosh, CG, Griffin JFT. Experimental infection model for Johne's disease in sheep. Infect Immun. (2005) 73:5603–11. 10.1128/IAI.73.9.5603-5611.2005 16113277 PMC1231139

[10] de Silva K Begg D Carter N Taylor D Di Fiore L Whittington R. The early lymphocyte proliferation response in sheep exposed to *Mycobacterium avium* subsp. paratuberculosis comprared to infection status. Immunobiology. (2010) 215:12–25. 10.1016/j.imbio.2009.01.014 19264377

[11] Fernández M Benavides J Sevilla IA Fuertes M Castaño P Delgado L. Experimental infection of lambs with C and S-type strains of *Mycobacterium avium* subspecies *paratuberculosis*: immunological and pathological findings. Vet Res. (2014) 45:5. 10.1186/1297-9716-45-5 24428881 PMC3897920

[12] Bastida F Juste RA. Paratuberculosis control: a review with a focus on vaccination. J Immune Based Ther Vaccines. (2011) 9:8. 10.1186/1476-8518-9-8 22035107 PMC3222599

[13] Juste R Pérez V. Control of paratuberculosis in sheep and goats. Vet Clin Food Anim. (2011) 27:127–38. 10.1016/j.cvfa.2010.10.020 21215897

[14] Eppleston J Reddacliff L Windsor P Links I Whittington R. Preliminary observations on the prevalence of sheep shedding *Mycobacterium avium* subsp *paratuberculosis* after 3 years of a vaccination program for ovine Johne's disease. Aust Vet J. (2005) 83:637–8. 10.1111/j.1751-0813.2005.tb13279.x 16255289

[15] Juste RA Alonso-Hearn M Molina E Geijo M Vazquez P Sevilla IA. Significant reduction in bacterial shedding and improvement in milk production in dairy farms after the use of a new inactivated paratuberculosis vaccine in a field trial. BMC Res Notes. (2009) 2:233. 10.1186/1756-0500-2-233 19930604 PMC2788577

[16] Reddacliff L Eppleston J Windsor P Whittington R Jones S. Efficacy of a killed vaccine for the control of paratuberculosis in Australian sheep flocks. Vet Microbiol. (2006) 115:77–90. 10.1016/j.vetmic.2005.12.021 16459030

[17] Sweeney RW Whitlock RH Bowersock TL Cleary DL Meinert TR Habecker PL. Effect of subcutaneous administration of a killed *Mycobacterium avium* subsp *paratuberculosis* vaccine on colonization of tissues following oral exposure to the organism in calves. Am J Vet Res. (2009) 70:493–7. 10.2460/ajvr.70.4.493 19335105

[18] Alonso-Hearn M Molina E Geijo M Vazquez P Sevilla IA Garrido JM. Immunization of adult dairy cattle with a new heat-killed vaccine is associated with longer productive life prior to cows being sent to slaughter with suspected paratuberculosis. J Dairy Sci. (2012) 95:618–29. 10.3168/jds.2009-2860 22281327

[19] Gwozdz JM Thompson KG Manktelow BW Murray A West DM. Vaccination against paratuberculosis of lambs already infected experimentally with *Mycobacterium avium* subspecies *paratuberculosis*. Aust Vet J. (2000) 78:560–6. 10.1111/j.1751-0813.2000.tb11902.x 10979513

[20] Begg DJ Griffin JFT. Vaccination of sheep against *M.* paratuberculosis: Immune parameters and protective efficacy. Vaccine. (2005) 23:4999–5008. 10.1016/j.vaccine.2005.05.031 15992970

[21] Mercier P Brémaud I Gautier MP. Vaccination of kids under one month of age with a killed vaccine and reduction in the frequency of faecal shedding of *Mycobacterium avium* subspecies *paratuberculosis*. Small Rumin Res. (2014) 121:425–33. 10.1016/j.smallrumres.2014.09.007

[22] de Silva K Plain KM Begg DJ Purdie AC Whittington RJ. CD4^+^ T-cells, γδ T-cells and B-cells are associated with lack of vaccine protection in *Mycobacterium avium* subspecies *paratuberculosis* infection. Vaccine. (2015) 33:149–55. 10.1016/j.vaccine.2014.10.082 25444806

[23] Dhand NK Eppleston J Whittington RJ Windsor PA. Changes in prevalence of ovine paratuberculosis following vaccition with Gudair®: results of a longitudinal study conducted over a decade. Vaccine. (2016) 34:5107–13. 10.1016/j.vaccine.2016.08.064 27614780

[24] Watkins C Schock A May L Denham S Sales J Welch L. Assessing virulence of vaccine strains of *Mycobacterium avium* subspecies *paratuberculosis* in a calf model. Vet Microbiol. (2010) 146:63–9. 10.1016/j.vetmic.2010.04.017 20472374

[25] Begg DJ Dhungyel O Naddi A Dhand NK Plain KM de Silva K. The immunogenicity and tissue reactivity of *Mycobacterium avium* subsp *paratuberculosis* inactivated whole cell vaccine is dependent on the adjuvant used. Heliyon. (2019) 5:e01911. 10.1016/j.heliyon.2019.e01911 31249894 PMC6584770

[26] Aucouturier J Dupuis L Ganne V. Adjuvants designed for veterinary and human vaccines. Vaccine. (2001) 19:2666–72. 10.1016/S0264-410X(00)00498-9 11257407

[27] van der Heijden PHJ Bokhout BA Bianchi ATJ Scholten JW Stok W. Separate application of adjuvant and antigen: the effect of a water-in-oil emulsion on the splenic plaque-forming cell response to sheep red blood cells in mice. Immunobiol. (1986) 171:143–54. 10.1016/S0171-2985(86)80023-7 3519438

[28] Danielsson R Eriksson H. Aluminium adjuvants in vaccines- a way to modulate the immune response. Semin Cell Dev Biol. (2021) 115:3–9. 10.1016/j.semcdb.2020.12.008 33423930

[29] Li X Xing R Xu C Liu S Qin Y Li K. Immunostimulatory effect of chitosan and quaternary chitosan: a review of potential vaccine adjuvants. Carbohydr Polym. (2021) 264:118050. 10.1016/j.carbpol.2021.118050 33910752

[30] Garrido JM Vazquez P Molina E Plazaola JM Sevilla IA Geijo MV. Paratuberculosis vaccination causes only limited cross-reactivity in the skin test for diagnosis of bovine tuberculosis. PLoS ONE. (2013) 8:e80985. 10.1371/journal.pone.0080985 24303029 PMC3841166

[31] Serrano M Elguezabal N Sevilla IA Geijo MV Molina E Arrazuria R. Tuberculosis detection in paratuberculosis vaccinated calves: New alternatives against interference. PLoS ONE. (2017) 12:e0169735. 10.1371/journal.pone.0169735 28072845 PMC5224860

[32] Pérez de Val B Nofrarías M López-Soria S Garrido JM Vordermeier HM Villareal-Ramos B Martín M Puentes E Juste RA Domingo M. Effects of vaccination against paratuberculosis on tuberculosis in goats: diagnostic interferences and cross-protection. BMC Vet Res. (2012) 8:191. 10.1186/1746-6148-8-191 23072619 PMC3514378

[33] Serrano M Elguezabal N Sevilla IA Geijo MV Molina E Juste RA. Preliminary results indicate that inactivated vaccine against paratuberulosis could modify the course of experimental *Mycobacterium bovis* infection in calves. Front Vet Sci. (2017) 4:175. 10.3389/fvets.2017.00175 29094040 PMC5651274

[34] Garrido JM Sevilla IA Beltrán-Beck B Minguijón E Ballesteros C Galindo RC. Protection against tuberculosis in Eurasian wild boar vaccinated with heat-inactivated Mycobacterium bovis. PloS ONE. (2011) 6:e24905. 10.1371/journal.pone.0024905 21935486 PMC3173485

[35] Arteche-Villasol N Benavides J Espinosa J Vallejo R Royo M Ferreras MC. Optimized *in vitro* isolation of different subpopulation of immune cells from peripheral blood and comparative techniques for generation of monocyte-derived macrophages in small ruminants. Vet Immunol Immunopathol. (2020) 230:110131. 10.1016/j.vetimm.2020.110131 33129192

[36] Delgado L Juste RA Muñoz M Morales S Benavides J Ferreras MC. Differences in the peripheral immune response between lambs and adult ewes experimentally infected with *Mycobacterium avium* suspecies *paratuberculosis*. Vet Immunol Immunopathol. (2012) 145:23–31. 10.1016/j.vetimm.2011.10.005 22070826

[37] Vidal E Arrieta-Villegas C Grasa M Mercader I Domingo M Pérez de Val B. Field evaluation of the efficacy of Mycobacterium bovis BCG vaccine against tuberculosis in goats. BMC Vet Res. (2017) 13:252. 10.1186/s12917-017-1182-5 28818102 PMC5561642

[38] Roy Á Infantes-Lorenzo JA Blázquez JC Venteo A Mayoral FJ Domínguez M. Temporal analysis of the interference caused by paratuberculosis vaccination on the tuberculosis diagnostic tests in goats. Prev Vet Med. (2018) 156:68–75. 10.1016/j.prevetmed.2018.05.010 29891147

[39] Lybeck KR Storset AK Djønne B Valheim M Olsen I. Faecal shedding detected earlier than immune responses in goats naturally infected with *Mycobacterium avium* subsp. paratuberculosis. Res Vet Sci. (2011) 91:32–9. 10.1016/j.rvsc.2010.08.012 20869736

[40] Arteche-Villasol N Gutiérrez-Expósito D Vallejo R Espinosa J Elguezabal N Ladero-Auñon I. Early response of monocyte-derived macrophages from vaccinated and non-vaccinated goats against *in vitro* infection with *Mycobacterium avium* subsp. paratuberculosis. Vet Res. (2021) 52:69. 10.1186/s13567-021-00940-y 33980310 PMC8117269

[41] Hines II ME Turnquist SE Ilha MRS Rajeev S Jones AL Whittington L. Evaluation of novel oral vaccine candidates and validation of a caprine model of Johne's disease. Front Cell Infect Microbiol. (2014) 4:26. 10.3389/fcimb.2014.00026 24624365 PMC3941644

[42] Plain KM Marsh IB Waldron AM Galea F Whittington AM Saunders VF. High-throughput direct fecal PCR assay for detection of *Mycobacterium avium* subsp. *paratuberculosis* in sheep and cattle. J Clin Microbiol. (2014) 52:745–57. 10.1128/JCM.03233-13 24352996 PMC3957769

[43] Rodríguez-Lázaro D D'Agostino M Herrewegh A Pla M Cook N Ikonomopoulos J. Real-time PCR-based methods for detection of *Mycobacterium avium* subsp. *paratuberculosis* in water and milk. Int J Food Microbiol. (2005) 101:93–104. 10.1016/j.ijfoodmicro.2004.09.005 15878410

[44] Adúriz JJ Juste RA Cortabarria N. Lack of mycobactin dependence of mycobacteria isolated on Middlebrook 7H11 from clinical cases of ovine paratuberculosis. Vet Microbiol. (1995) 45:211–7. 10.1016/0378-1135(95)00037-B 7571372

[45] Sevilla IA Garrido JM Molina E Geijo MV Elguezabal N Vázquez P. Development and evaluation of a novel multicopy-element-targeting triplex PCR for detection of *Mycobacterium avium* subsp. *paratuberculosis* in feces. Appl Environ Microbiol. (2014) 80:3757–68. 10.1128/AEM.01026-14 24727272 PMC4054133

[46] Delgado L García Marín JF Muñoz M Benavides J Juste RA García-Pariente C. Pathological findings in young and adult sheep following experimental infection with 2 different doses of *Mycobacterium avium* subspecies *paratuberculosis*. Vet Pathol. (2013) 50:857–66. 10.1177/0300985813476066 23390077

[47] Rosseels V Huygen K. Vaccination against paratuberculosis. Expert Rev Vaccines. (2008) 7:817–32. 10.1586/14760584.7.6.817 18665779

[48] Gupta S Singh SV Singh M Chaubey KK Karthik K Bhatia AK. Vaccine approaches for the “therapeutic management” of *Mycobacterium avium* subspecies *paratuberculosis* infection in domestic livestock. Vet Q. (2019) 39:143–52. 10.1080/01652176.2019.1667042 31524561 PMC6831026

[49] Krüger C Köhler H Liebler-Tenorio EM. Sequential development of lesions 3, 6, 9, and 12 months after experimental infection of goat kids with *Mycobacterium avium* subsp *paratuberculosis*. Vet Pathol. (2015) 52:276–90. 10.1177/0300985814533804 24829286

[50] Juste RA García Marín JF Peris B Sáez de Ocáriz C Badiola JJ. Experimental infection of vaccinated and non-vaccinated lambs with Mycobacterium *paratuberculosis*. J Comp Path. (1994) 110:185–94. 10.1016/S0021-9975(08)80189-2 8040384

[51] Faisal SM. Chen JW Yan F Chen TT Useh NM Yan W. Evaluation of a Mycobacterium avium subsp paratuberculosis leuD mutant as a vaccine candidate against challenge in a caprine model. Clin Vaccine Immunol. (2013) 20:572–81. 10.1128/CVI.00653-12 23408524 PMC3623397

[52] Corpa JM Pérez V García Marín JF. Differences in the immune responses in lambs and kids vaccinated against paratuberculosis, according to the age of vaccination. Vet Microbiol. (2000) 77:475–85. 10.1016/S0378-1135(00)00332-1 11118732

[53] Storset AK Hasvold HJ Valheim M Brun-Hansen H Berntsen G Whist SK. Press CMcL, Holstad G, Larsen HJS. Subclinical paratuberculosis in goats following experimental infection: an immunological and microbiological study. Vet Immunol Immunopathol. (2001) 80:271–87. 10.1016/S0165-2427(01)00294-X 11457480

[54] Vazquez P Garrido JM Juste RA. Specific antiody and interferon-gamma responsen associated with immunopathological forms of bovine paratuberculosis in slaughtered Friesian cattle. PLoS ONE. (2013) 8:e64568. 10.1371/journal.pone.0064568 23724062 PMC3665815

[55] Koets A Ravesloot L Ruuls R Dinkla A Eisenberg S Lievaart-Peterson K. Effects of age and environment on adaptive immune responses to *Mycobacterium avium* subsp. *paratuberculosis* (MAP) vaccination in dairy goats in relation to *paratuberculosis* control strategies. Vet Sci. (2019) 6:62. 10.3390/vetsci6030062 31266267 PMC6789810

[56] Pérez V Tellechea J Badiola JJ Gutiérrez M García Marín JF. Relation between serologic response and pathologic findings in sheep with naturally acquired paratuberculosis. Am J Vet Res. (1997) 58:799–803. Available online at: https://www.avma.org/Pages/home.aspx 9256958

[57] Pollock JM McNair J Welsh MD Girvin RM Kennedy HE Mackie DP. Immune response in bovine tuberculosis. Tuberculosis. (2001) 81:103–7. 10.1054/tube.2000.0258 11463230

[58] Waters WR Miller JM Palmer MV Stabel JR Jones DE Koistinen KA. Early induction of humoral and cellular immune responses during experimental *Mycobacterium avium* subsp. *paratuberculosis* infection of calves. Infect Immun. (2003) 71:5130–8. 10.1128/IAI.71.9.5130-5138.2003 12933856 PMC187349

[59] Begg DJ de Silva K Carter N Plain KM Purdie A. Whittington RJ. Does a Th1 over Th2 dominancy really exist in the early stages of Mycobacterium avium subspecies paratuberculosis infections? Immunobiology. (2011) 216:840–6. 10.1016/j.imbio.2010.12.004 21281979

[60] Pooley HB Begg DJ Plain KM Whittington RJ Purdie AC de Silva K. The humoral immune response is essential for successful vaccine protection against *paratuberculosis* in sheep. BMC Vet Res. (2019) 15:223. 10.1186/s12917-019-1972-z 31266499 PMC6604481

[61] Storset AK Berg I Djønne B. Evaluation of the gamma interferon test for diagnosis of paratuberculosis in goats. Vet Immunol Immunopathol. (2005) 107:87–94. 10.1016/j.vetimm.2005.03.015 15885801

[62] Denis M Wedlock DN Buddle BM. Ability of T cell subsets and their soluble mediators to modulate the replication of *Mycobacterium bovis* in bovine macrophages. Cell Immunol. (2004) 232:1–8. 10.1016/j.cellimm.2005.01.003 15922710

[63] Gillan S O'Brien R Hughes AD Griffin JFT. Identification of immune parameters to differentiate disease states among sheep infected with *Mycobacterium avium* subsp. paratuberculosis. Clin Vaccine Immunol. (2010) 17:108–17. 10.1128/CVI.00359-09 19923568 PMC2812105

[64] Frie MC Sporer KRB Kirkpatrick BW Coussens PM. T and B cell activation profiles from cows with and without Johne's disease in response to *in vitro* stimulation with *Mycobacterium avium* subspecies *paratuberculosis*. Vet Immunol Immunopathol. (2017) 193–4:50–6. 10.1016/j.vetimm.2017.10.005 29129227

[65] Stabel JR Bannantine JP. Divergent antigen-specific cellular immune responses during asymptomatic subclinical and clincal states of disease in cows naturally infected with *Mycobacterium avium* subsp. paratuberculosis. Infec Immun. (2020) 88:e00650–19. 10.1128/IAI.00650-19 31611273 PMC6921652

[66] Arrieta-Villegas C Perálvarez T Vidal E Puighibet Z Moll X Canturri A. Efficacy of parenteral vaccination against tuberculosis with heat-inactivated Mycobacterium bovis in experimentally challenged goats. PLoS ONE. (2018) 13:e0196948. 10.1371/journal.pone.0196948 29742150 PMC5942842

[67] Kurade NP Tripathi BN Rajukumar K Parihar NS. Sequential development of histologic lesions and their relationship with bacterial isolation, fecal shedding, and immune responses during progressive stages of experimental infection of lambs with *Mycobacterium avium* subsp. Paratuberculosis. Vet Pathol. (2004) 41:378–87. 10.1354/vp.41-4-378 15232138

[68] Taniguchi Y Sakakibara S Fujihara M Yagi A Fujiyoshi S. The association between detection of *Mycobacterium avium* subsp. *paratuberculosis* DNA in feces and histopathological classification. J Vet Med Sci. (2020) 82:541–5. 10.1292/jvms.18-0724 32161236 PMC7273589

[69] Whittington RJ Fell S Walker D McAllister S Marsh I Sergeant E. Use of pooled fecal culture for sensitive and economic detection of *Mycobacterium avium* subsp. *paratuberculosis* infection in flocks of sheep. J Clin Microbiol. (2000) 38:2550–6. 10.1128/JCM.38.7.2550-2556.2000 10878042 PMC86966

[70] Mortier RA Barkema HW Orsel K Wolf R De Buck J. Shedding patterns of dairy calves experimentally infected with *Mycobacterium avium* subspecies *paratuberculosis*. Vet Res. (2014) 45:71. 10.1186/s13567-014-0071-1 25224905 PMC4347591

[71] González J Geijo MV García-Pariente C Verna A Corpa JM Reyes LE. Histopathological classification of lesions associated with natural paratuberculosis infection in cattle. J Comp Path. (2005) 133:184–96. 10.1016/j.jcpa.2005.04.007 16045917

[72] Roy Á Risalde MA Bezos J Casal C Romero B Sevilla I. Response of goats to intramuscular vaccination with heat-killed *Mycobacterium bovis* and natural challenge. Comp Immunol Microbiol Infect Dis. (2018) 60:28–34. 10.1016/j.cimid.2018.09.006 30396427

[73] Muskens J van Zijderveld F Eger A Bakker D. Evaluation of the long-term immune response in cattle after vaccination against *paratuberculosis* in two Dutch dairy herds. Vet Microbiol. (2002) 86:269–78. 10.1016/S0378-1135(02)00006-8 11900960

[74] Thom M Howard C Villarreal-Ramos B Mead E Vordermeier M Hope J. Consequence of prior exposure to environmental mycobacteria on BCG vaccination and diagnosis of tuberculosis infection. Tuberculosis. (2008) 88:324–34. 10.1016/j.tube.2007.12.002 18329343

[75] Álvarez J de Juan L Bezos J Romero B Sáez JL Marqués S. Effect of paratuberculosis on the diagnosis of bovine tuberculosis in a cattle herd with a mixed infection using interferon-gamma detection assay. Vet Microbiol. (2009) 135:389–93. 10.1016/j.vetmic.2008.09.060 18986776

[76] Tewari D Hoving E Linscott R Martel E Lawrence J Wolfgang D. *Mycobacterium avium* subsp. *paratuberculosis* antibody response, fecal shedding, and antibody cross-reactivity to Mycobacterium bovis in M avium subsp *paratuberculosis*-infected cattle herds vaccinated against Johne's disease. Clin Vaccine Immunol. (2014) 21:698–703. 10.1128/CVI.00032-14 24623626 PMC4018888

[77] Jones GJ Steinbach S Sevilla IA Garrido JM Juste R Vordermeier HM. Oral vaccination of cattle with heat inactivated *Mycobacterium bovis* does not compromise bovine TB diagnostic tests. Vet Immunol Immunopathol. (2016) 182:85–8. 10.1016/j.vetimm.2016.10.010 27863556

[78] Coffman RL Sher A Seder RA. Vaccine adjuvants: Putting innate immunity to work. Immunity. (2010) 33:492–503. 10.1016/j.immuni.2010.10.002 21029960 PMC3420356

[79] Awate S Babiuk LA Mutwiri G. Mechanism of action of adjuvants. Front Immunol. (2013) 4:114. 10.3389/fimmu.2013.00114 23720661 PMC3655441

[80] van der Heijden EMDL Chileshe J Vernooij JCM Gortazar C Juste RA Sevilla I. Immune response profiles of calves following vaccination with live BCG and inactivated *Mycobacterium bovis* vaccine candidates. PLoS ONE. (2017) 12:e0188448. 10.1371/journal.pone.0188448 29155877 PMC5695775

[81] Vordermeier HM Dean GS Rosenkrands I Agger EM Andersen P Kaveh DA. Adjuvants induce distinct immunological phenotypes in a bovine tuberculosis vaccine model. Clin Vaccine Immunol. (2009)16:1443–8. 10.1128/CVI.00229-09 19641101 PMC2756844

[82] Klimka A Michels L Glowalla E Tosetti B Krönke M Krut O. Montanide ISA 71 VG is advantageous to Freund's adjuvant in immunization against *S. aureus* infection of mice. Scand J Immunol. (2015) 81:291–7. 10.1111/sji.12279 25689117

[83] Netea MG. Training innate immunity: the changing concept of immunological memory in innate host defence. Eur J Clin Invest. (2013) 43:881–4. 10.1111/eci.12132 23869409

