# Supplementary material for: Influence of Heterologous and Homologous Vaccines, and Their Components, on the Host Immune Response and Protection Against Experimental Caprine Paratuberculosis
Source: Front Vet Sci. 2022 Jan 5;8:744568. doi: 10.3389/fvets.2021.744568 (PMC8767014; doi:10.3389/fvets.2021.744568)
Supplement: Supplementary file 1 [file Data_Sheet_1.docx]

Supplementary Material

# Supplementary Data

| **Supplementary Table 1:** Flow cytometry analysis of lymphocyte subpopulations present in PBMCs isolated from whole blood at each time sampled (days post-vaccination: dpv). | | | | | | | |
| --- | --- | --- | --- | --- | --- | --- | --- |
| **Group** | **Time (dpv)** | **CD4 %** | **CD8 %** | **WC1 %** | **CD21 %** | **CD3 %** | **CD20 %** |
| NV | 0 | 33.67 ± 3.00 | 10.06 ± 3.48 | 21.88 ± 3.40 | 35.74 ± 1.36 | 14.90 ± 4.13 | 19.32 ± 4.95 |
|  | 30 | 35.04 ± 5.17 | 16.30 ± 4.17 | 30.85 ± 7.34 | 29.80 ± 2.82 | 25.65± 6.66 | 20.37± 5.25 |
|  | 60 | 22.16 ± 4.54 | 10.67 ± 2.46 | 19.85± 1.16 | 38.27 ± 12.88 | 24.17 ± 9.74 | 17.37 ± 6.32 |
|  | 90 | 31.04 ± 1.05 | 13.92 ± 1.87 | 23.52 ± 7.39 | 34.72 ± 7.73 | 19.40 ± 9.09 | 10.40 ± 2.30 |
|  | 120 | 33.29 ± 7.36 | 13.54 ± 4.09 | 31.08 ± 5.84 | 30.91 ± 5.43 | 18.58 ± 9.22 | 10.34 ± 5.09 |
|  | 150 | 24.38 ± 3.93 | 10.88 ± 5.68 | 24.65 ± 4.96 | 20.99 ± 4.75 | 16.73 ± 3.10 | 8.16 ± 1.88 |
|  | 190 | 25.53 ± 3.63 | 12.25 ± 5.02 | 23.56 ± 3.77 | 22.13 ± 4.44 | 16.56 ± 2.88 | 7.81 ± 1.59 |
| NVI | 0 | 29.88 ± 3.50 | 14.12 ± 2.10 | 29.76 ± 4.08 | 25.89 ± 6.06 | 22.02 ± 2.30 | 27.23 ± 3.29 |
|  | 30 | 35.19 ± 4.59 | 15.33 ± 3.27 | 37.15 ± 3.33 | 25.65 ± 8.41 | 31.67 ± 4.19 | 27.89 ± 2.53 |
|  | 60 | 23.84 ± 8.98 | 15.68 ± 7.69 | 31.86 ± 2.99 | 25.89 ± 11.45 | 23.21 ± 3.33 | 14.75 ± 3.28 |
|  | 90 | 29.59 ± 3.80 | 14.94 ± 5.10 | 38.97 ± 7.93 | 26.98 ± 5.42 | 30.61 ± 5.02 | 20.28 ± 5.96 |
|  | 120 | 31.97 ± 5.57 | 16.05 ± 6.11 | 41.52 ± 7.34 | 22.29 ± 7.82 | 21.14 ± 3.14 | 11.82 ± 2.60 |
|  | 150 | 27.13 ± 3.59 | 14.16 ± 4.53 | 27.05 ± 6.63 | 21.10 ± 4.78 | 16.26 ± 3.21 | 6.95 ± 2.14 |
|  | 190 | 26.78 ± 3.64 | 14.01 ± 4.14 | 24.83 ± 4.61 | 21.18 ± 4.25 | 15.06 ± 2.80 | 5.37 ± 1.63 |
| VS | 0 | 28.42 ± 5.22 | 23.02 ± 6.03 | 32.81 ± 8.20 | 30.98 ± 4.36 | 20.76 ± 2.16 | 27.08 ± 0.52 |
|  | 30 | 34.81 ± 3.03 | 16.24 ± 1.39 | 31.54 ± 5.55 | 30.12 ± 5.17 | 29.21 ± 5.25 | 22.13 ± 3.53 |
|  | 60 | 30.26 ± 3.95 | 16.44 ± 4.58 | 31.03 ± 3.37 | 28.21 ± 6.02 | 21.79 ± 8.59 | 12.94 ± 4.27 |
|  | 90 | 34.63 ± 2.45 | 15.58 ± 2.61 | 35.63 ± 5.63 | 24.00 ± 6.03 | 24.40 ± 3.00 | 15.03 ± 2.96 |
|  | 120 | 32.38 ± 3.21 | 19.66 ± 3.76 | 37.37 ± 6.64 | 22.62 ± 5.00 | 25.22 ± 2.33 | 13.64 ± 1.49 |
|  | 150 | 23.22 ± 1.94 | 15.35 ± 1.76 | 32.81 ± 9.08 | 12.69 ± 0.14 | 10.05 ± 4.82 | 6.60 ± 1.52 |
|  | 190 | 25.77 ± 1.75 | 13.87 ± 2.20 | 23.13 ± 10.31 | 13.21 ± 0.18 | 12.73 ± 1.73 | 4.70 ± 1.58 |
| VSI | 0 | 25.34 ± 4.91 | 19.82 ± 4.10 | 27.18 ± 9.79 | 30.23 ± 9.57 | 23.82 ± 7.01 | 29.35 ± 8.61 |
|  | 30 | 33.36 ± 8.99 | 15.74 ± 5.74 | 30.25 ± 1.90 | 29.43 ± 6.84 | 19.49 ± 1.91 | 17.86 ± 2.92 |
|  | 60 | 23.79 ± 6.65 | 13.93 ± 6.65 | 39.62 ± 7.14 | 27.76 ± 5.98 | 18.59 ± 3.62 | 9.06 ± 1.96 |
|  | 90 | 26.58 ± 5.58 | 15.99 ± 6.10 | 41.25 ± 1.18 | 23.23 ± 0.56 | 27.99 ± 5.55 | 18.51 ± 7.18 |
|  | 120 | 26.23 ± 5.79 | 15.61 ± 5.99 | 44.44 ± 4.92 | 23.59 ± 3.56 | 20.33 ± 5.37 | 10.92 ± 3.32 |
|  | 150 | 20.83 ± 2.97 | 12.87 ± 2.61 | 38.58 ± 5.92 | 17.79 ± 1.82 | 17.30 ± 3.11 | 4.52 ± 1.81 |
|  | 190 | 19.78 ± 1.52 | 12.13 ± 0.95 | 31.25 ± 12.34 | 16.74 ± 2.54 | 14.89 ± 2.23 | 3.23 1.36 |
| VH | 0 | 27.46 ± 8.06 | 20.42 ± 6.31 | 22.08 ± 2.20 | 38.33 ± 8.41 | 20.89 ± 3.18 | 26.60 ± 5.11 |
|  | 30 | 36.49 ±1.11 | 22.89 ± 2.30 | 31.63 ± 5.54 | 31.00 ± 6.96 | 29.50 ± 1.43 | 20.39 ± 3.13 |
|  | 60 | 31.30 ± 1.60 | 14.73 ± 1.40 | 25.55 ± 5.37 | 32.75 ± 9.47 | 23.29 ± 4.38 | 14.40 ± 4.85 |
|  | 90 | 37.60 ± 1.98 | 17.11 ± 4.87 | 35.58 ± 1.55 | 28.26 ± 5.25 | 23.58 ± 2.22 | 13.97 ± 1.82 |
|  | 120 | 36.16 ± 3.27 | 18.44 ± 1.60 | 29.36 ± 12.41 | 24.99 ± 8.05 | 29.70 ± 3.83 | 18.26 ± 5.40 |
|  | 150 | 27.22 ± 0.41 | 13.45 ± 3.87 | 31.44 ± 6.69 | 16.39 ± 2.61 | 14.52 ± 0.22 | 6.28 ± 1.10 |
|  | 190 | 28.09 ± 0.71 | 14.33 ± 3.90 | 27.71 ± 5.13 | 17.21 ± 2.83 | 14.35 ± 0.10 | 6.02 ± 1,06 |
| VHI | 0 | 26.03 ± 10.26 | 22.61 ± 10.21 | 19.67 ± 2.77 | 36.27 ± 6.86 | 20.55 ± 2.42 | 25.81 ± 2.16 |
|  | 30 | 31.58 ± 4.83 | 23.84 ± 6.49 | 30.37 ± 4.26 | 30.37 ± 7.53 | 29.28 ± 10.59 | 24.54 ± 10.78 |
|  | 60 | 31.43 ± 3.11 | 16.98 ± 3.33 | 25.72 ± 5.27 | 33.76 ± 10.30 | 9.73 ± 8.06 | 8.35 ± 3.24 |
|  | 90 | 37.06 ± 1.77 | 18.23 ± 7.27 | 33.90 ± 6.44 | 28.29 ± 3.29 | 27.21 ± 7.10 | 18.10 ± 5.80 |
|  | 120 | 29.91 ± 4.35 | 13.71 ± 1.13 | 30.23 ± 9.31 | 26.18 ± 8.17 | 26.23 ± 6.78 | 17.58 ± 3.32 |
|  | 150 | 26.19 ± 3.77 | 14.99 ± 1.81 | 30.00 ± 7.89 | 23.40 ± 6.63 | 16.25 ± 3.59 | 6.36 ± 1.15 |
|  | 190 | 25.61 ± 3.32 | 14.73 ± 1.74 | 27.74 ± 7.45 | 22.73 ± 6.32 | 14.25 ± 3.29 | 4.37 ± 1.20 |
| StrSI | 0 | 34.35 ± 9.76 | 19.79 ± 8.01 | 25.47 ± 17.28 | 40.61 ± 21.02 | 23.04 ± 6.74 | 27.90 ± 6.88 |
|  | 30 | 34.01 ± 4.83 | 24.81 ± 6.49 | 26.56 ± 4.26 | 29.57 ± 7.53 | 16.25 ± 10.59 | 10.91 ± 10.78 |
|  | 60 | 27.98 ± 6.16 | 20.42 ± 8.64 | 28.03 ± 3.90 | 30.48 ± 1.25 | 21.95 ± 2.22 | 12.98 ± 2.99 |
|  | 90 | 30.82 ± 4.63 | 14.96 ± 3.75 | 34.81 ± 11.94 | 26.25 ± 1.69 | 21.31 ± 2.65 | 13.64 ± 1.00 |
|  | 120 | 31.21 ± 3.63 | 18.53 ± 2.88 | 41.56 ± 6.77 | 16.73 ± 2.53 | 24.90 ± 4.16 | 15.86 ± 2.95 |
|  | 150 | 27.83 ± 4.76 | 17.24 ± 2.68 | 31.72 ± 3.32 | 19.00 ± 2.37 | 15.11 ± 1.07 | 4.48 ± 0.68 |
|  | 190 | 24.84 ± 4.00 | 12.51 ± 1.48 | 12.82 ± 3.61 | 16.53 ± 3.51 | 11.39 ± 0.47 | 2.96 ± 0.35 |
| StrHI | 0 | 31.86 ± 9.76 | 21.02 ± 7.83 | 23.73 ± 8.30 | 31.26 ± 8.64 | 18.48 ± 4.08 | 26.15 ± 5.60 |
|  | 30 | 34.29 ± 4.17 | 20.24 ± 9.18 | 34.05 ± 8.93 | 33.32 ± 4.56 | 21.94 ± 4.76 | 17.57 ± 2.46 |
|  | 60 | 26.87 ± 9.78 | 11.31 ± 2.07 | 34.19 ± 17.25 | 30.88 ± 9.29 | 21.13 ± 3.47 | 11.80 ± 5.40 |
|  | 90 | 31.08 ± 8.44 | 11.19 ± 0.97 | 31.29 ± 12.11 | 29.01 ± 9.71 | 22.02 ± 4.89 | 11.58 ± 5.49 |
|  | 120 | 31.69 ± 4.56 | 15.52 ± 2.88 | 31.00 ± 13.12 | 27.33 ± 16.10 | 22.09 ± 6.30 | 17.89 ± 6.41 |
|  | 150 | 21.96 ± 3.51 | 14.50 ± 3.59 | 30.90 ± 15.07 | 21.11 ± 7.72 | 20.11 ± 5.02 | 8.03 ± 2.96 |
|  | 190 | 21.42 ± 3.46 | 14.46 ± 3.23 | 30.39 ± 15.27 | 20.55 ± 6.57 | 18.02 ± 4.90 | 5.62 ± 2.74 |
| AdjSI | 0 | 27.61 ± 1.90 | 16.86 ± 6.39 | 17.55 ± 6.00 | 27.17 ± 9.28 | 27.76 ± 7.03 | 33.78 ± 9.26 |
|  | 30 | 28.80 ± 1.86 | 12.51 ± 0.50 | 17.33 ± 12.12 | 19.64 ± 5.65 | 10.34 ± 5.02 | 3.00 ± 1.98 |
|  | 60 | 23.86 ± 5.54 | 15.83 ± 8.79 | 33.41 ± 5.17 | 40.00 ± 9.99 | 21.08 ± 3.41 | 14.50 ± 1.33 |
|  | 90 | 26.00 ± 3.99 | 15.43 ± 3.84 | 37.62 ± 6.20 | 24.95 ± 4.10 | 16.73 ± 8.46 | 10.34 ± 6.31 |
|  | 120 | 28.96 ± 1.80 | 19.01 ± 2.03 | 40.43 ± 4.81 | 23.14 ± 7.68 | 22.06 ± 9.17 | 13.19 ± 5.03 |
|  | 150 | 19.51 ± 1.88 | 13.28 ± 1.67 | 29.28 ± 6.29 | 19.26 ± 4.72 | 15.35 ± 2.88 | 4.46 ± 1.29 |
|  | 190 | 18.21 ± 1.57 | 10.64 ± 1.77 | 13.63 ± 2.35 | 18.32 ± 3.98 | 8.70 ± 0.38 | 2.11 ± 0.45 |
| AdjHI | 0 | 28.41 ± 1.28 | 16.94 ± 5.58 | 23.19 ± 6.05 | 30.18 ± 1.73 | 25.06 ± 0.08 | 33.12 ± 8.48 |
|  | 30 | 34.98 ± 4.33 | 15.67 ± 4.35 | 34.10 ± 11.67 | 31.31 ± 7.40 | 23.21 ± 12.39 | 18.79 ± 15.36 |
|  | 60 | 24.84 ± 1.44 | 13.88 ± 4.27 | 36.94 ± 6.05 | 34.01 ± 2.18 | 19.09 ± 6.08 | 10.81 ± 2.83 |
|  | 90 | 27.74 ± 4.17 | 14.97 ± 4.58 | 36.51 ± 7.44 | 33.93 ± 2.91 | 23.81 ± 5.85 | 15.12 ± 4.32 |
|  | 120 | 29.84 ± 1.65 | 18.20 ± 4.97 | 42.69 ± 5.05 | 23.47 ± 0.34 | 27.37 ± 1.02 | 18.03 ± 1.48 |
|  | 150 | 22.49 ± 1.36 | 14.26 ± 1.84 | 28.18 ± 4.21 | 25.93 ± 5.64 | 12.52 ± 3.10 | 3.73 ± 1.42 |
|  | 190 | 21.78 ± 1.41 | 14.09 ± 2.13 | 27.47 ± 4.24 | 25.26 ± 5.65 | 15.26 ± 1.41 | 3.97 ± 1.31 |
| Non-vaccinated and non-infected (NV); Non-vaccinated and infected (NVI); Silirum^®^ vaccinated and non-infected (VS); Silirum^®^ vaccinated and infected (VSI); HIMB vaccinated and non-infected (VH); HIMB vaccinated and infected (VHI); *Map* 316F Silirum^®^ strain immunized and infected (StrSI); *Mbv* 1403 HIMB strain immunized and infected (StrHI); Montanide^™^ Silirum^®^ adjuvant immunized and infected (AdjSI); Montanide^™^ HIMB adjuvant immunized and infected (AdjHI). | | | | | | | |

| **Supplementary Table 2:** Flow cytometry analysis of lymphocyte subpopulations in distal ileum from all vaccination groups. | | | | | | |
| --- | --- | --- | --- | --- | --- | --- |
| **Group** | **CD4 %** | **CD8 %** | **WC1 %** | **CD21 %** | **CD3 %** | **CD20 %** |
| NV | **6.21 ± 2.02^a^**** | 2.98 ± 1.00 | 0.71 ± 0.35 | 35.84 ± 9.26 | 2.91 ± 0.89 | 2.94 ± 1.51 |
| NVI | 8.02 ± 3.68 | 3.72 ± 1.54 | 0.48 ± 0.16 | 39.44 ± 3.45 | 2.00 ± 0.35 | 1.98 ± 0.58 |
| VS | **7.79 ± 0.87^a^*** | 3.74 ± 0.65 | 0.91 ± 0.24 | 38.23 ± 9.71 | 3.98 ± 1.96 | 3.16 ± 1.30 |
| VSI | **7.80 ± 1.61^a^*** | 3.94 ± 1.63 | 1.06 ± 0.85 | 36.82 ± 8.11 | 2.50 ± 0.57 | 1.67 ± 0.60 |
| VH | **6.16 ± 1.21^a^*** | 3.35 ± 1.19 | 1.09 ± 0.59 | 31.79 ± 5.08 | 3.14 ±0.40 | 3.44 ± 1.21 |
| VHI | 12.74 ± 5.08 | 5.50 ± 1.71 | 1.27 ± 0.81 | 45.59 ± 10.05 | 3.00 ± 0.86 | 2.19 ± 0.61 |
| StrSI | 8.62 ± 2.07 | 5.49 ± 2.39 | 0.90 ± 0.28 | 39.33 ± 8.92 | 2.53 ± 0.35 | 1.80 ± 0.50 |
| StrHI | 8.31 ± 1.78 | 4.57 ± 1.20 | 0.95 ± 0.47 | 43.51 ± 3.78 | 2.60 ± 0.65 | 2.79 ± 1.33 |
| AdjSI | 11.21 ± 3.59 | 4.92 ± 1.84 | 1.12 ± 0.72 | 44.15 ± 6.46 | 2.76 ± 1.40 | 1.90 ± 0.98 |
| AdjHI | 16.03 ± 3.82 | 5.78 ± 2.12 | 1.42 ± 0.98 | 46.35 ± 9.18 | 3.19 ± 0.54 | 2.79 ± 1.48 |
| Results are expressed as mean values and standard deviations of percentage of positive cells for each surface marker. Significant differences are expressed as * (*P* < 0.05), ** (*P* < 0.01), *** (*P* < 0.001) and **** (*P* < 0.0001).  Non-vaccinated and non-infected (NV); Non-vaccinated and infected (NVI); Silirum^®^ vaccinated and non-infected (VS); Silirum^®^ vaccinated and infected (VSI); HIMB vaccinated and non-infected (VH); HIMB vaccinated and infected (VHI); *Map* 316F Silirum^®^ strain immunized and infected (StrSI); *Mbv* 1403 HIMB strain immunized and infected (StrHI); Montanide^™^ Silirum^®^ adjuvant immunized and infected (AdjSI); Montanide^™^ HIMB adjuvant immunized and infected (AdjHI).  ^a^ Significantly different from AdjHI | | | | | | |

| **Supplementary Table 3:** Flow cytometry analysis of lymphocyte subpopulations in jejunal Peyer’s patches from all vaccination groups. | | | | | | |
| --- | --- | --- | --- | --- | --- | --- |
| **Group** | **CD4 %** | **CD8 %** | **WC1 %** | **CD21 %** | **CD 3%** | **CD 20%** |
| NV | **7.85 ± 1.97^a^*** | 7.48 ± 3.98 | 1.44 ± 0.51 | 24.63 ± 7.17 | 7.65 ± 2.22 | 4.09 ± 1.37 |
| NVI | 9.43 ± 5.23 | **12.08 ± 5.33^b^*** | 1.11 ± 0.45 | 23.18 ± 3.55 | 8.00 ± 2.58 | 2.46 ± 0.84 |
| VS | **7.79 ± 2.91^a^*** | 5.03 ± 1.94 | 1.97 ± 2.00 | 29.82 ± 11.34 | **7.14 ± 5.29^e^*** | 4.80 ± 3.95 |
| VSI | **7.20 ± 2.17^a^**** | 8.65 ± 5.40 | 1.62 ± 1.45 | 20.73 ± 7.47 | 7.17 ± 4.39 | 2.70 ± 0.93 |
| VH | **6.41 ± 1.46^a^*** | **6.17 ± 1.27^c^*** | 2.03 ± 0.26 | 30.71 ± 2.69 | 5.95 ± 1.68 | 4.83 ± 2.02 |
| VHI | 14.12 ± 3.08 | **13.44 ± 5.91 ^b^**** | 2.30 ± 0.69 | 25.31 ± 8.98 | 8.54 ± 4.07 | **3.38 ± 0.90 ^e^*** |
| StrSI | **6.75 ± 2.40^a^*** | 6.35 ± 3.20 | 1.74 ± 1.40 | 31.87 ± 6.70 | 3.15 ± 1.22 | 1.88 ± 0.53 |
| StrHI | 11.42 ± 2.81 | **9.77 ± 4.54^c^*** | 1.72 ± 0.82 | 26.64 ± 6.19 | 5.94 ± 2.71 | 3.10 ± 0.58 |
| AdjSI | 9.44 ± 4.23 | 6.89 ± 5.71 | 1.29 ± 1.27 | 30.95 ± 2.39 | 4.11 ± 2.39 | 1.99 ± 0.84 |
| AdjHI | 16.13 ± 4.96 | **12.28 ± 5.17 ^b^*** | 1.92 ± 1.04 | 22.86 ± 3.55 | 8.55 ± 3.55 | **3.21 ± 1.16 ^e^*** |
| Results are expressed as mean values and standard deviations of percentage of positive cells for each surface marker. Significant differences are expressed as * (*P* < 0.05), ** (*P* < 0.01), *** (*P* < 0.001) and **** (*P* < 0.0001).  Non-vaccinated and non-infected (NV); Non-vaccinated and infected (NVI); Silirum^®^ vaccinated and non-infected (VS); Silirum^®^ vaccinated and infected (VSI); HIMB vaccinated and non-infected (VH); HIMB vaccinated and infected (VHI); *Map* 316F Silirum*^®^* strain immunized and infected (StrSI); *Mbv* 1403 HIMB strain immunized and infected (StrHI); Montanide^™^ Silirum^®^ adjuvant immunized and infected (AdjSI); Montanide^™^ HIMB adjuvant immunized and infected (AdjHI).  ^a^ Significantly different from AdjHI  ^b^ Significantly different from VS  ^c^ Significantly different from VHI  ^e^ Significantly different from StrSI | | | | | | |

| **Supplementary Table 4:** Flow cytometry analysis of lymphocyte subpopulations in mesenteric lymph node from all vaccination groups. | | | | | | |
| --- | --- | --- | --- | --- | --- | --- |
| **Group** | **CD4 %** | **CD8 %** | **WC1 %** | **CD21 %** | **CD20 %** | **CD3 %** |
| NV | **9.52 ± 5.85^a^**** | 5.00 ± 1.78 | 0.95 ± 0.29 | 40.96 ± 6.93 | 5.34 ± 1.54 | 3.73 ± 0.20 |
| NVI | 12.36 ± 2.31 | 8.33 ± 2.65 | 0.43 ± 0.25 | 50.01 ± 4.49 | 5.90 ± 1.38 | 4.17 ± 1.30 |
| VS | **10.82 ± 4.15 ^a^*** | 6.06 ± 2.94 | 0.87 ± 0.54 | 40.74 ± 11.04 | 5.44 ± 1.02 | 3.90 ± 1.26 |
| VSI | **9.88 ± 3.78 ^a^**** | 5.90 ± 3.15 | 1.16 ± 1.06 | 42.83 ± 4.91 | 4.52 ± 2.34 | 3.36 ± 1.49 |
| VH | **11.51 ± 6.36 ^a^*** | 5.49 ± 2.76 | 1.15 ± 0.47 | 40.68 ± 5.51 | 6.39 ± 1.98 | 4.57 ± 1.28 |
| VHI | 11.72 ± 5.28 | 5.41 ± 2.85 | 0.80 ± 0.42 | 50.39 ± 8.21 | 4.04 ± 0.99 | 3.13 ± 1.04 |
| StrSI | **9.67 ± 2.38 ^a^**** | 6.46 ± 3.07 | 1.02 ± 0.40 | 47.48 ± 5.39 | 5.09 ± 2.52 | 3.14 ± 0.66 |
| StrHI | **9.41 ± 2.01 ^a^**** | 5.75 ± 0.44 | 0.64 ± 0.59 | 47.69 ± 4.72 | 4.10 ± 1.72 | 3.73 ± 1.17 |
| AdjSI | **10.41 ± 3.61 ^a^*** | 5.39 ± 1.35 | 1.05 ± 0.89 | 42.39 ± 5.16 | 3.37 ± 1.42 | 2.33 ± 0.66 |
| AdjHI | 18.83 ± 4.84 | 10.48 ± 2.98 | 1.42 ± 0.32 | 46.80 ± 11.61 | 6.67 ± 1.66 | 4.81 ± 0.84 |
| Results are expressed as mean values and standard deviations of percentage of positive cells for each surface marker. Significant differences are expressed as * (*P* < 0.05), ** (*P* < 0.01), *** (*P* < 0.001) and **** (*P* < 0.0001).  Non-vaccinated and non-infected (NV); Non-vaccinated and infected (NVI); Silirum^®^ vaccinated and non-infected (VS); Silirum^®^ vaccinated and infected (VSI); HIMB vaccinated and non-infected (VH); HIMB vaccinated and infected (VHI); *Map* 316F Silirum^®^ strain immunized and infected (StrSI); *Mbv* 1403 HIMB strain immunized and infected (StrHI); Montanide^™^ Silirum^®^ adjuvant immunized and infected (AdjSI); Montanide^™^ HIMB adjuvant immunized and infected (AdjHI).  ^a^ Significantly different from AdjHI | | | | | | |
